# Comprehensive evaluation of artificial intelligence-empowered approaches for protein–aptamer complex prediction

**DOI:** 10.1093/bib/bbag206

**Published:** 2026-05-04

**Authors:** Jiani Zhao, Kha Tram, Hongbin Yan, Yifeng Li

**Affiliations:** Department of Computer Science, Brock University, 1812 Sir Isaac Brock Way, St. Catharines, L2S 3A1 Ontario, Canada; Cytodiagnostics Inc., 919 Fraser Dr Unit 11, Burlington, L7L 4X8 Ontario, Canada; Department of Chemistry, Brock University, 1812 Sir Isaac Brock Way, St. Catharines, L2S 3A1 Ontario, Canada; Department of Biological Sciences, Brock University, 1812 Sir Isaac Brock Way, St. Catharines, L2S 3A1 Ontario, Canada; Department of Computer Science, Brock University, 1812 Sir Isaac Brock Way, St. Catharines, L2S 3A1 Ontario, Canada; Department of Biological Sciences, Brock University, 1812 Sir Isaac Brock Way, St. Catharines, L2S 3A1 Ontario, Canada

**Keywords:** protein–aptamer complexes, AI-based structure prediction, benchmarking, MD simulations, aptamer specificity

## Abstract

Drug discovery is a time-consuming, expensive, and high-risk process. Recent advances in artificial intelligence (AI) have enabled major breakthroughs in small-molecule and protein therapeutics. However, AI-driven design of aptamer drugs remains largely unexplored. Aptamers are short (15–100 nt) single-stranded DNAs or RNAs that exhibit high binding affinity, high specificity, and low immunogenicity, making them promising candidates for disease (such as cancer) therapeutics. Compared with protein–ligand or protein–protein systems, protein–aptamer complexes are under-represented in public structural databases, and aptamers themselves are highly flexible and relatively large molecules. These characteristics present distinct challenges for AI-based structural modeling. Here, we systematically evaluate recent AI frameworks, including AlphaFold3, Chai-1, Boltz-2, and RoseTTAFold2NA, along with a template-based approach, in predicting protein–aptamer complex structures and estimating binding free energies. We establish an independent benchmark to assess their performance in structural accuracy, stability, and energetic consistency. This study provides a foundation for the application of AI in aptamer drug design and offers a reference framework for future research in nucleic-acid therapeutics and biomolecular modeling.

## Introduction

Molecular biology and pharmaceutical science fundamentally rely on understanding and modulating molecular interactions that underlie biological functions. Effective therapeutics generally possess the ability to specifically recognize their biological targets, form stable binding interfaces, and maintain structural stability under physiological conditions. Small molecules and antibodies have long achieved significant success, but these therapeutics are still limited by chemical diversity, target accessibility, and manufacturing complexity [1–4]. Nucleic acids, including aptamers, have emerged as a class of promising therapeutics owing to their programmability, high specificity, and relative ease of chemical synthesis [5, 6]. Aptamers are short single-stranded DNA or RNA molecules that fold into defined 3D conformations and bind their targets with high affinity and selectivity [7]. Their binding characteristics resemble those of antibodies but usually do not cause immunogenicity, and exhibit greater tunability and stability under specific conditions. The traditional approach to aptamer identification using systematic evolution of ligands by exponential enrichment is labor-intensive and time-consuming, requiring multiple rounds of experimental screening and optimization [5, 6]. The complexity of this process has motivated the development of *in silico* computational strategies to predict aptamer structures and their interactions with target proteins, improving the efficiency of discovery [8, 9].

Early efforts in molecular structure prediction primarily relied on physics-based paradigms that describe molecular interactions through energy functions and force fields [10, 11]. These approaches provided qualitative mechanistic insights but were constrained by simplified energy scoring functions, flexibility at active binding sites, precision of force field parameters, limited sampling efficiency, and high computational cost, which restricted accuracy and scalability [12, 13]. As experimental data accumulate, data-driven approaches were developed to make use of known sequence–structure associations. For example, 3dRNA/DNA [14] is a template-based approach to predict tertiary structures of RNAs and DNAs. Machine learning approaches have also long been considered to predict protein and nucleic acid secondary structures as classification problems [15], and their tertiary structures as regression problems [16]. Protein–protein interaction prediction is often modeled as a two-class classification problem [17]. Different from template-based approaches that mainly consider explicit mapping between sequences and structures, machine learning approaches are able to leverage informative representation learning and capture intrinsic nonlinear data relationships. The recent development of deep learning techniques, particularly with graph neural networks [18–22], variational autoencoders [23, 24], multi-objective optimization and deep reinforcement learning [25–28], attention mechanisms [29] and Transformers [30, 31], and diffusion models [32–35], enabled big data-driven embedding representations and modeling of complex molecular relationships. The advent of AlphaFold2 [36] marked a major advance, achieving near-atomic accuracy in protein structure prediction and establishing a general framework for deep learning-based molecular modeling. Inspired by AlphaFold2, RoseTTAFold [37] utilized a 3-track neural network to predict protein folding structures and protein–protein complex structures. RoseTTAFold has been extended to RoseTTAFold2NA [38] for the modeling of protein and nucleic acid complexes. Building upon AlphaFold2’s foundation, AlphaFold3 [39] extended predictive capability to a broad range of molecular types, including nucleic acids and ligands, and their interactions through a diffusion-based architecture. Subsequent developments continued to expand this paradigm toward increasingly diverse and integrated biomolecular assemblies. AlphaFold3 did not share its code with the community when their work was first published [40]. Academic and corporate researchers re-implemented AlphaFold3 based on the available pseudocode and extended its features. Derivative models, such as Chai-1 [41], retain the core AlphaFold3 design while introducing mechanisms to incorporate experimental constraints. Boltz-1 [42], built upon AlphaFold3, optimizes the model architecture and computational efficiency, while its successor Boltz-2 [43] further introduces controllability features and an affinity-prediction module for estimating the binding affinity between proteins and small molecules. Despite these advances, most of these AlphaFold-inspired methods remain general in design and have not been systematically evaluated for their performance on protein–aptamer complexes.

Recent advances in deep learning-based structure prediction frameworks have substantially broadened the scope of biomolecular modeling, motivating the need for systematic benchmarks to assess model performance and applicability. Benchmarking efforts in protein complex prediction have focused predominantly on protein–protein and protein–ligand systems. For example, Yin *et al*. [44] systematically evaluated AlphaFold2 for protein–protein complexes, and Leemann *et al*. [45] developed an automated framework on protein–ligand benchmarking within the CASP15 experiment. For protein–nucleic acid complexes, the recently released CASP16 [46] expanded its evaluation protocol to include blind assessments of these systems. However, the CASP16 assessment includes a limited number of protein–nucleic acid targets, and many lack the corresponding Protein Data Bank (PDB) [47] identifiers. This absence makes it difficult to determine whether any of the entries involve protein–aptamer systems, and the associated data cannot be used in our dataset. Other benchmarks, such as ProNASet [48], have been proposed for evaluating protein–nucleic acid predictions using root mean square deviation (RMSD), template modeling (TM)-score, and local distance difference test (LDDT), although their scope remains narrower than that of CASP16. As ProNASet focuses exclusively on general protein–nucleic acid complexes and does not include any protein–aptamer systems, it is not directly comparable with, nor suitable for inclusion in, our benchmark. By contrast, systematic benchmarking for protein–aptamer complex prediction remain limited, with existing studies largely restricted to preliminary analyses and lacking both breadth and depth. Although CASP16 protocols could potentially be applied, these assessments primarily emphasize geometric accuracy and provide limited characterization of energetic consistency or interfacial stability. Among the few existing studies, Ochoa *et al*. [49] evaluated AlphaFold3 on a mixed dataset of aptamer and nucleic acid systems containing only a small subset of protein–aptamer complexes. Their evaluation relied mainly on RMSD and Matthews correlation coefficients to quantify base-pairing and base-stacking accuracy. While these results offer a useful reference, the dataset composition and limited metrics make it difficult to draw definitive conclusions regarding model reliability for protein–aptamer complex prediction.

Here, we present the first systematic and independent benchmark of state-of-the-art artificial intelligence (AI)-based structure prediction frameworks on protein–aptamer complexes, evaluating both structural accuracy and energetic consistency. We assembled a dedicated benchmark set by collecting all experimentally determined protein–aptamer complexes deposited in the PDB after the AlphaFold3 training cutoff, excluding closely related variants from the same study and entries whose aptamer sequences contain modified residues (e.g. those annotated as “X”), and constructing mismatched pairs under strict constraints to ensure valid nonbinding controls. Each framework was evaluated across multiple dimensions, including interface accuracy, conformational stability, and energetic consistency. In addition, we compared the number of interfacial hydrogen bonds (H-bonds) between predicted and experimental structures to assess the ability of each model to capture key interaction determinants. This study delineates the strengths and limitations of current AI frameworks for modeling protein–aptamer interactions and provides a reference foundation for future refinement of deep-learning approaches for aptamer modeling and therapeutic design.

## Background

In this study, we compare threegeneral-purpose structure prediction models (AlphaFold3, Chai-1, and Boltz-2), one specialized model (RoseTTAFold2NA) from the Rosetta family, and a representative traditional approach (3dRNA/DNA) for the prediction of protein–aptamer complexes. A brief overview is provided below to explain the working principles of these models, and their main methodological characteristics are summarized in Table 1.

**Table 1:** Summary of the structure prediction models evaluated in this study.

	AlphaFold3	Chai-1	Boltz-2	RoseTTAFold2NA	3dRNA/DNA
Modeling targets	Prediction of biomolecular structures and interactions involving proteins, nucleic acids, ligands, ions, and covalent modifications			Specialized prediction of protein–nucleic acid complexes and nucleic acid structures	Prediction of RNA and DNA tertiary structures
Primary input	Multiple biomolecular sequences			Protein and nucleic acid sequences	Nucleic acid sequences, with optional secondary-structure input
Modeling strategy	PairFormer-based trunk with diffusion-based all-atom structure generation			Rigid-frame and torsion-angle-based modeling	Template-based SSE decomposition and 3D assembly
Output metrics	pLDDT, PAE, pTM, ipTM			Predicted distogram, per-residue LDDT, PAE	Energy-based ranking (3dRNAscore/3dDNAscore)
Special features	Unified diffusion-based framework for joint modeling of diverse biomolecular complexes	User-specified constraints (e.g. inter-chain contacts or covalent bonds)	User-specified constraints and protein-small-molecule affinity prediction	Unified modeling of proteins and nucleic acids for direct complex prediction	Optional constraints and user-selectable loop building/refinement

### AlphaFold3

AlphaFold2 achieved high-precision prediction of protein structures and their interactions, substantially advancing computational approaches for protein modeling and design. Building on this progress, AlphaFold3 (AF3) broadens this paradigm to encompass a wider range of biomolecular systems by introducing a substantially redesigned diffusion-based generative architecture that unifies the prediction of proteins, nucleic acids, ligands, ions, and covalently modified residues under a single integrated framework [39]. By directly operating on raw atomic coordinates and coarse-grained token representations, the diffusion module in AF3 removes the dependence on amino-acid-specific reference frames and side-chain torsion angles used in AlphaFold2. This design eliminates the need for torsion-based residue parameterizations and stereochemical constraint terms, thereby enabling the model to capture the full chemical complexity of general ligands [39]. During training, a Transformer-based denoiser conditioned on features extracted from the main trunk is trained to remove Gaussian noise from the positions of all heavy atoms. At inference, the model begins from random noise and progressively refines structures through recurrent denoising to generate the final structure.

During optimization, AF3 minimizes a composite loss function that integrates diffusion, structural, and confidence objectives. The overall loss is defined as:


(1) \begin{align*} \mathcal{L}_{\mathrm{loss}} ={} &\ \alpha_{\mathrm{confidence}} \cdot (\mathcal{L}_{\mathrm{plddt}} + \mathcal{L}_{\mathrm{pde}}+\mathcal{L}_{\mathrm{resolved}} + \alpha_{\mathrm{pae}} \cdot \mathcal{L}_{\mathrm{pae}}) \nonumber \\ & +\alpha_{\mathrm{diffusion}} \cdot\mathcal{L}_{\mathrm{diffusion}}+ \alpha_{\mathrm{distogram}} \cdot \mathcal{L}_{\mathrm{distogram}},\end{align*}

where each $\alpha $ denotes a weighting coefficient for its corresponding loss term. These loss terms are described below.

#### Confidence losses

**Predicted local distance difference test (pLDDT).** The per-atom confidence score is defined to capture local structural accuracy through an LDDT-based metric that includes distances from all atoms to polymer residues. The loss is defined as:


(2) \begin{align*}& \mathcal{L}_{\mathrm{plddt}} = -\frac{1}{N_{\mathrm{atom}}} \sum_{l} \sum_{b=1}^{50} \mathrm{lddt}_{l}^{b} \log p_{l}^{b},\end{align*}

where


(3) \begin{align*}& \mathrm{lddt}_{l} = \sum_{m \in R} \frac{1}{4} \sum_{c \in \{0.5, 1, 2, 4\}} d_{lm} < c.\end{align*}

Here $\mathrm{lddt}_{l}^{b}$ denotes the discretized (binned) representation of $\mathrm{lddt}_{l}$; $d_{lm}$ is the distance between atom $l$ and atom $m$ in the mini-rollout prediction; $R$ represents polymer atoms near atom $l$ in the ground-truth (GT) structure; and $p_{l}^{b}$ is the predicted probability for bin $b$ obtained via softmax.

**Predicted alignment error (PAE) and predicted distance error (PDE).** Within the model, a pairwise confidence between atoms is introduced to reflect confidence across molecular interfaces and atomic interactions. PAE estimates the error in the relative position and orientation between two tokens in the predicted structure, whereas PDE predicts the error in absolute distances between atoms. PAE and PDE’s training losses follow an identical formulation, defined as:


(4) \begin{align*}& \mathcal{L}_{\mathrm{pae}},\ \mathcal{L}_{\mathrm{pde}} = -\frac{1}{N_{\mathrm{token}}^{2}}\sum_{i,j} \sum_{b=1}^{64} e_{ij}^{b} \log p_{ij}^{b}.\end{align*}

For PAE, the PAE $e_{ij}$ corresponds to the Euclidean distance between the predicted and ground truth coordinates of the token $j$ after aligning the two structures on token $i$, whereas for PDE, it represents the absolute difference between the predicted and ground truth pairwise distances, with the predicted distance defined as the distance between representative token atoms $i$ and $j$ in the mini-rollout structure. Here $e_{ij}^{b}$ denotes the binned representation of $e_{ij}$.

A single value for each pair of tokens can be expressed as:


(5) \begin{align*}& \mathrm{PAE}_{ij},\ \mathrm{PDE}_{ij} = \sum_{b=1}^{64} \Delta_{b} p_{ij}^{b},\end{align*}

where $\Delta _{b}$ denotes the centers of the distance bin.

**Experimentally resolved prediction.** The model includes an auxiliary objective that predicts whether each atom ($y_{l}$) is experimentally resolved. The loss is defined as:


(6) \begin{align*}& \mathcal{L}_{\mathrm{resolved}} = -\frac{1}{N_{\mathrm{atom}}}\sum_{l} \sum_{b=1}^{2} y_{l}^{b} \log p_{l}^{b}.\end{align*}

#### Diffusion losses

The overall diffusion objective comprises a weighted mean squared error (MSE) loss, an auxiliary bond-length loss, and a smooth LDDT-based structural loss:


(7) \begin{align*}& \mathcal{L}_{\mathrm{diffusion}} = \frac{(\hat{t}^{2} + \sigma_{\mathrm{data}}^{2})}{(\hat{t} + \sigma_{\mathrm{data}})^{2}}\cdot(\mathcal{L}_{\mathrm{MSE}} + \alpha_{\mathrm{bond}} \cdot \mathcal{L}_{\mathrm{bond}}) + \mathcal{L}_{\mathrm{smooth}{\_}\mathrm{lddt}},\end{align*}

where $\hat{t}$ denotes the sampled noise level, with $\sigma _{\mathrm{data}}$ reflecting the variance of the training data, and $\alpha _{\mathrm{bond}}$ applied across different training stages.

**MSE.** The MSE loss is applied to the denoised structure output from the diffusion module, and is defined as:


(8) \begin{align*}& \mathcal{L}_{\mathrm{MSE}} = \frac{1}{3} \, \mathop{\mathrm{mean}}\limits_{l}\left( w_{l} \, \| \vec{\mathbf{x}}_{l} -\vec{\mathbf{x}}_{l}^{\mathrm{GT_{aligned}}} \|^{2} \right)\kern-2pt,\end{align*}

where $w_{l}$ denotes the weighting factors applied to nucleotide and ligand atoms, and $\vec{\mathbf{x}}_{l}^{\mathrm{GT_{aligned}}}$ corresponds to the ground truth coordinates $\vec{\mathbf{x}}_{l}^{\mathrm{GT}}$ rigidly aligned with denoised structure $\vec{\mathbf{x}}_{l}$.

**Bond-length.** To maintain correct distances for bonded ligands, a bond-length loss is applied during fine-tuning:


(9) \begin{align*}& \mathcal{L}_{\mathrm{bond}} = \mathop{\mathrm{mean}}\limits_{(l,m)\in\mathcal{B}}\left(\left\| \vec{\mathbf{x}}_{l} - \vec{\mathbf{x}}_{m}\right\|-\left\| \vec{\mathbf{x}}_{l}^{\mathrm{GT}}-\vec{\mathbf{x}}_{m}^{\mathrm{GT}}\right\|\right)^{2},\end{align*}

where $\mathcal{B}$ denotes the set of atom pairs forming covalent linkages between ligand atoms and their parent chains.

**Smooth LDDT.** The model further employs a sigmoid-based smoothing scheme to generate continuous distance-difference scores over atom pairs. For details of the smooth LDDT-based loss, refer to AF3 Algorithm 27 in [39].

#### Structural loss

**Distogram prediction.** In addition to confidence objectives, the structural objective predicts binned inter-token distances using the same formulation as AlphaFold2 [36, 39], with the loss defined as:


(10) \begin{align*} \mathcal{L}_{\mathrm{distogram}} ={} & -\frac{1}{N_{\mathrm{res}}^{2}} \sum_{i,j} \sum_{b=1}^{64} y_{ij}^{b} \log p_{ij}^{b},\end{align*}

where $y_{ij}^{b}$ denotes the one-hot representation of binned residue distances.

### Chai-1

Within the AF3 architectural paradigm, Chai-1 retains the same foundational design while introducing extensions that provide additional functionality. To enhance representation quality, an additional input track incorporates residue-level embeddings from a large protein language model, as described in the Chai-1 technical report [41], thereby improving single sequence capability and reducing the dependency on multiple sequence alignments (MSAs). In parallel, the architecture integrates constraint features that represent experimentally derived geometric information, including pocket, contact, and docking constraints. These constraints can be specified from prior knowledge or inferred from experimental observations, providing auxiliary spatial guidance for structure prediction. As the constraint functionalities and the single sequence mode were not employed in this study, the corresponding mechanisms are not further discussed.

### Boltz-2

Building upon the AF3 and Boltz-1 frameworks, Boltz-2 introduced controllability mechanisms and an affinity prediction module [42] that enables estimation of binding affinity between small molecules and proteins. The controllability mechanisms include method, template, and contact/pocket conditioning, each introducing distinct contextual constraints during structure generation. Method conditioning specifies the experimental modality (e.g. X-ray crystallography), guiding the prediction toward structural distributions consistent with the selected experimental conditions. Template conditioning allows external template structures to be incorporated during prediction, while contact/pocket conditioning enables distance constraints to be defined based on experimental data or prior knowledge. For affinity estimation, the model employs a PairFormer backbone followed with dual prediction heads for binding likelihood and continuous affinity prediction on a logarithmic scale, following the formulation described in the Boltz-2 report. As the constraint and affinity prediction functionalities were not employed in this study, the corresponding mechanisms are not further discussed.

**Diffusion loss.** The model uses a diffusion-based loss similar to AF3, defined as:


(11) \begin{align*}& \mathcal{L}_{\mathrm{diffusion}} = \frac{(\hat{t}^{2} + \sigma_{\mathrm{data}}^{2})}{(\hat{t} \cdot \sigma_{\mathrm{data}})^{2}} \cdot(\mathcal{L}_{\mathrm{MSE}} + \alpha_{\mathrm{bond}} \cdot \mathcal{L}_{\mathrm{bond}}) + \mathcal{L}_{\mathrm{smooth}{\_}\mathrm{lddt}},\end{align*}

where the weighting of the diffusion term follows the Elucidating the Design space of diffusion Model formulation [42, 50], replacing the $(\hat{t}^{2} + \sigma _{\mathrm{data}}^{2})/(\hat{t} \boldsymbol{+} \sigma _{\mathrm{data}})^{2}$ weighting used in AF3 (Equation 7) [39].

### RoseTTAFold2NA

RF2NA extends the RoseTTAFold framework to achieve unified modeling of protein–nucleic acid complexes. For nucleic acid components, the model adopts an all-atom representation analogous to that used in AlphaFold for amino acids [38], in which each nucleotide is represented by a rigid frame centered on the phosphate group together with a set of torsion angles. During training, full-atom coordinates are generated kinematically from the phosphate group along the torsional chain to reconstruct the complete structure. The total loss is defined as:


(12) \begin{align*} \mathcal{L} ={} &\ w_{\mathrm{seq}} \cdot \mathrm{seq} + w_{\mathrm{6D}} \cdot \mathrm{6D} + w_{\mathrm{str}} \cdot \mathrm{str} \nonumber \\ & + w_{\mathrm{tors}} \cdot \mathrm{tors} + w_{\mathrm{err}} \cdot \mathrm{err},\end{align*}

where $\mathrm{seq}$ denotes the masked amino acid recovery loss, and $\mathrm{6D}$ represents the six-dimensional distogram loss that encodes inter-residue geometric relationships. The term $\mathrm{str}$ corresponds to the structure loss, which combines backbone and all-atom Frame Aligned Point Error (FAPE) components across structure layers. The term $\mathrm{tors}$ denotes the torsion-angle prediction loss averaged over all layers, and $\mathrm{err}$ represents the pLDDT confidence loss. The coefficients $w$ weight the relative contributions of each component in the overall objective.

A subsequent fine-tuning stage is applied following initial training to improve stereochemical quality. The corresponding loss is defined as:


(13) \begin{align*} \mathcal{L}_{\mathrm{finetune}} ={} &\ \mathcal{L} + w_{\mathrm{LJ}} \cdot \mathrm{LJ} + w_{\mathrm{hbond}} \cdot \mathrm{hbond} \nonumber \\ & + w_{\mathrm{geom}} \cdot \mathrm{geom} + w_{\mathrm{pairerr}} \cdot \mathrm{pairerr},\end{align*}

where $\mathrm{LJ}$ and $\mathrm{hbond}$ correspond to the Lennard–Jones and H-bond energy terms of the final structure, respectively. The $\mathrm{geom}$ term constrains bond lengths and angles around peptide or phosphodiester linkages, and is implemented as a linear penalty following the formulation used in RoseTTAFold2; and $\mathrm{pairerr}$ represents the predicted residue-pair error.

### 3dRNA/DNA

3dRNA/DNA is a template-based framework for tertiary structure prediction of RNAs and DNAs from sequence and secondary structure inputs. Each target is decomposed into secondary structure elements (SSEs), which are assigned 3D templates from a DNA template library. When suitable templates are unavailable, substitute fragments are generated using the bi-residue or distance-geometry algorithms [51]. The selected templates are subsequently assembled into a complete 3D model through iterative superposition guided by shared base pairs using the Kabsch algorithm [52]. Following assembly, the structure undergoes energy minimization or simulated-annealing Monte Carlo refinement to improve chain continuity and local geometric accuracy. As each SSE may correspond to multiple compatible templates, the procedure yields several candidate conformations, and the overall accuracy of the assembled structures largely depends on identification of appropriate templates for each SSE.

## Methods

### Datasets

We curated a benchmark set of 11 protein–aptamer complexes from the PDB that were released after the AF3 training cutoff date (30 September 2021). For consistency across models, the AF3 training cutoff was adopted as the benchmark cutoff, because the training data used for Chai-1 and RF2NA were restricted to earlier PDB releases. In contrast, Boltz-2 was trained on all PDB entries released up to 1 June 2023. Consequently, six of the 11 complexes (7LRI, 7SZU, 7V5N, 7ZKO, 7ZQS, and 8D29) fall within its training set, whereas the remaining five (8BW5, 8TFD, 8TQS, 8ZBF, and 9GXH) were released after this date and therefore excluded from its training set. To minimize redundancy, a single representative structure was selected from groups of PDB entries originating from the same study (e.g. 7LRI, 7LRM, 7LRX, 7LRY, 7LSK), as these entries correspond to closely related variants of the same complex. The resulting 11 entries represent the complete set of experimentally determined protein–aptamer complexes currently available in the PDB that satisfy these selection criteria (Table 2). Some PDB entries contain aptamer sequences with modified residues (e.g. annotated as “X”), which could not be encoded by the evaluated models and were therefore excluded from the benchmark dataset. These complexes may be incorporated in further analyses to expand the dataset if suitable preprocessing methods become available.

**Table 2:** List of PDB entries used in our dataset.

PDB ID	Aptamer length (nt)	Target	Category	Type	Protein length (aa)	Function	Organism
7LRI	35	HIV-1 reverse transcriptase	Enzymes	DNA	429, 555	Reverse transcriptase	HIV-1
7SZU	67	BL3-6 Fab	RNA-binding proteins	RNA	224	Antigen binding	Mus Musculus
7V5N	24	Bevacizumab Fab	Fragment antigen-binding	DNA	214, 231	Antigen binding	Homo Sapiens
8D29	34	Human antibody Fab fragment	Fragment antigen-binding	RNA	214, 229	Antigen binding	Homo Sapiens
8TQS	30	Human alpha thrombin	Enzymes	DNA	41, 259	Thrombin for blood clotting	Homo Sapiens
7ZKO	15	Human alpha thrombin	Enzymes	DNA	36, 259	Thrombin for blood clotting	Homo Sapiens
8BW5	41	Human alpha thrombin	Enzymes	DNA	36, 259	Thrombin for blood clotting	Homo Sapiens
7ZQS	51	Human transferrin receptor 1	Transferrins	DNA	760	Iron uptake via receptor-mediated endocytosis	Homo Sapiens
8TFD	20	SARS-CoV-2 nucleocapsid	RNA-binding proteins	DNA	130	RNA packaging and viral replication	SARS-CoV-2
8ZBF	40	SARS-CoV-2 nucleocapsid	RNA-binding proteins	DNA	135	RNA packaging and viral replication	SARS-CoV-2
9GXH	15	Nanobody bound to TBA G-quadruplex	Nanobody	DNA	121	Antigen binding	Lama Glama

Note: Aptamer lengths are reported in nucleotides (nt), whereas protein lengths are reported in amino acids (aa). Protein lengths with two values correspond to the two protein chains in the complex.

### Models

All structure prediction models were employed in their latest available versions (accessed May 2025). AF3 (v3.0.1, released 23 January 2025) was deployed through Docker, whereas Chai-1 (v0.6.1, released March 2025), Boltz-2 (v2.2.0, released July 2025), and RF2NA (v0.2, released April 2023) were run locally. Predictions from 3dRNA/DNA were generated using its official web server (http://biophy.hust.edu.cn/new/3dRNA).

### Experimental setup

Protein and aptamer sequences extracted from Biological Assembly 1 of each PDB entry were used as model inputs and defined the positive (native) set. To assess model specificity, a mismatched negative set (hereafter, negative set) was constructed by pairing each protein sequence with the aptamer from the next complex (i.e. protein1 with aptamer2, protein2 with aptamer3,..., protein11 with aptamer1), resulting in a total of 11 negative protein–aptamer pairs. To ensure that the negative pairs represented genuine nonbinding relationships, aptamers originating from the same or homologous proteins were not reassigned to those proteins or to other members of the same family (e.g. 8TQS, 7ZKO, and 8BW5 correspond to human $\alpha $-thrombin and were not cross-paired).

To further challenge model specificity and reduce the possibility of nonspecific binding in mismatched pairs, an additional shuffled-sequence negative-control set (shuffle set) was constructed for AF3-specific analyses by randomly permuting each native aptamer sequence while preserving sequence length and overall nucleotide composition, resulting in one shuffled-sequence control pair for each of the 11 benchmark complexes. A shuffled sequence was retained only if at least 80% of nucleotide positions differed from the native sequence, thereby ensuring substantial disruption of sequence order and potential recognition motifs. A fixed random seed was used to ensure reproducibility. In the main analyses, structure prediction for the positive, negative, and shuffle sets was performed using only protein and aptamer sequences, with no explicit ion input from the corresponding experimental structures. This input regime was used to maintain a consistent model input across control groups, as explicit ion specification is not uniformly supported across models. A separate ion-inclusion analysis was carried out only for the positive and mismatched negative sets, as described in Section Effect of ions on protein–aptamer complexes. Unless otherwise stated, Biological Assembly 1 of each PDB entry was used as the GT structure, with all hydrogen atoms, water molecules, ions, ligands, and other nonprotein and non-nucleic-acid components removed.

AF3, Chai-1, and Boltz-2 were evaluated under an AF3-matched sampling regime, with five seeds and five diffusion samples per seed, yielding 25 predictions per protein–aptamer complex. For Chai-1 and Boltz-2, the number of recycling steps was set to 10 to match the AF3 inference regime, whereas all other parameters were left at their default values. As RF2NA is not diffusion-based, 25 independent predictions were generated for each complex. In all cases, each model used its internally generated MSA.

Predictions were ranked by model confidence, and the top two predictions for each complex were selected for molecular dynamics (MD) simulations. For RF2NA, ranking was performed using the mean per-residue predicted LDDT.

System topologies were generated with CHARMM-GUI [53–55] and converted for GROMACS [56]. A rectangular water box was constructed with a 20 Å buffer fitted to the protein dimensions. NaCl was added as the sole ion type at pH 7 to provide a standardized ionic environment and system neutralization. All simulations were performed using the CHARMM36m force field at 300 K for 20 ns.

For aptamer-only predictions, aptamer sequences extracted from the 11 protein–aptamer complexes were used as inputs for AF3, Chai-1, Boltz-2, and RF2NA, and 3dRNA/DNA was additionally evaluated for this task. The same procedures and parameter settings as those used for the protein–aptamer predictions were applied to AF3, Chai-1, Boltz-2, and RF2NA, yielding 25 predictions per aptamer, from which the top-ranked structure was selected using the same criteria.

For 3dRNA/DNA, four seeds were generated using the Assemble procedure and one using Optimize, each producing five predictions, yielding 25 structures per aptamer. Secondary structures were obtained using RNAFold [57]. To prevent template reuse, the PDB entry of the corresponding protein–aptamer complex was provided through the “Exclude PDB” option during prediction; e.g. 7LRI was entered when predicting the isolated aptamer derived from 7LRI. The bi-residue loop-building procedure and AMBER-based energy minimization were applied. Predictions were found to be largely insensitive to the number of seeds, and we therefore selected the lowest-scoring structure from the two procedures as the final model. The resulting aptamer-only structures were then prepared for MD simulations.

### Evaluation metrics

Model performance was evaluated on two related tasks: prediction of protein–aptamer complexes and prediction of aptamer-only structures. For protein–aptamer complexes, the two highest-ranked predictions for each PDB entry (see Section Experimental setup) were subjected to MD simulations. Structural and stability metrics—including the radius of gyration (Rg), RMSD, and H-bond occupancies obtained from MD trajectories and HBPLUS analysis—were derived from these simulations. Unless otherwise noted, the prediction with the lower Molecular Mechanics Generalized Born Surface Area (MM/GBSA) binding free energy ($\Delta G_{\mathrm{bind}}$) was selected as the representative model. Aptamer-only predictions were evaluated analogously using the top-ranked structure for each target, with analysis restricted to MD-derived Rg and RMSD.

For the following metrics, higher iLDDT values were considered preferable, whereas the remaining metrics were interpreted relative to the GT reference.

#### Protein–aptamer complexes

**Binding free energy.**  $\Delta G_{\mathrm{bind}}$ was computed from MD trajectories using the gmx_MMPBSA [58]. Calculations were performed within the MM/GBSA formalism using the GB model defined by igb = 5 and saltcon = 0.150. Production MD simulations were performed with dt = 0.002 ps and nstxout-compressed = 5000, corresponding to one trajectory frame every 10 ps. MM/GBSA calculations were performed on frames 1001-2000, corresponding to the 10.01–20.00 ns interval and totaling 1000 frames. Before free-energy calculations, trajectories were corrected for periodic boundary conditions and recentered on the complex. No explicit entropy calculation was performed; accordingly, the reported $\Delta G_{\mathrm{bind}}$ values correspond to MM/GBSA estimates without entropy correction. Dielectric constants were kept at the default gmx_MMPBSA settings (intdiel = 1.0, extdiel = 78.5). To assess the stability of the MM/GBSA estimates, the trajectory window was further divided into four consecutive 2.5 ns subwindows, and $\Delta G_{\mathrm{bind}}$ was computed independently for each segment. Consistency across these subwindow estimates was used to assess the stability of the MM/GBSA binding free energy and to identify potential fluctuations within the analyzed trajectory.

For MM/GBSA group selection, the protein was defined as the receptor, and a custom-defined Aptamer group was used as the ligand. The default GROMACS index groups did not provide a single predefined group covering the full aptamer; nucleotide residue groups were therefore merged according to residue identity. Systems containing THY were treated as DNA, with nucleotide groups merged into THY, GUA, CYT, and ADE, whereas systems containing URA were treated as RNA, with groups merged into GUA, URA, ADE, and CYT. The resulting nucleic-acid group was renamed Aptamer and used as the ligand in subsequent MM/GBSA analysis.

**Pocket occupancy during MD.** Pocket occupancy (PO) was used as an atom-based measure to quantify whether the aptamer remained associated with the binding pocket during MD simulations. Protein–aptamer recognition typically involves an extended interface rather than in a canonical deep small pocket. The binding pocket was therefore defined on the basis of direct protein–aptamer contacts. The pocket was first defined on the GT complex in PyMOL as protein residues having at least one heavy atom within 4.5 Å of any aptamer heavy atom. Since residue numbering was not consistent across structures generated by different prediction models, corresponding residues in each predicted structure were assigned by sequence-based mapping to the GT sequence rather than by direct reference to GT residue numbering. Specifically, the protein sequence of the GT structure was globally aligned to the protein sequence of each predicted model, and residues aligned to the GT pocket residues were taken as the corresponding pocket residues in the predicted structure. These mapped pocket residues were then used as the gmx select group for MD analysis. For each frame, gmx select was used to identify aptamer atoms within 4.5 Å of this pocket-residue group, thereby yielding the number of aptamer atoms occupying the pocket over time. PO was calculated as


\begin{align*} & {\mathrm{PO}}(t)=\frac{N_{\mathrm{atom},t}}{N_{\mathrm{atom,total}}}, \end{align*}

where $N_{\mathrm{atom},t}$ is the number of aptamer atoms occupying the pocket at frame $t$, and $N_{\mathrm{atom,total}}$ is the total number of aptamer atoms in the corresponding complex. Thus, PO reflects the fraction of total aptamer atoms that remain in contact with the binding pocket during MD.

**Interface local distance difference test (iLDDT).** iLDDT scores were computed using OpenStructure [59] with default settings. Each predicted complex was compared with the corresponding experimental structure to assess local interface accuracy.

**HBPLUS hydrogen-bond analysis.** Interfacial hydrogen-bonds (H-bonds) between the protein and the aptamer were quantified using HBPLUS [60] (v.3.06). HBPLUS output was subsequently parsed to retain only H-bonds formed between protein atoms and aptamer atoms. Default criteria were used: minimum angle of 90$^\circ $ for D–H–A, H–A–AA, and D–A–AA, and maximum distance of 2.5 Å for H–A and 3.9 Å for D–A.

**Hydrogen bonds during MD.** Interfacial H-bonds between the protein and the aptamer were identified from MD trajectories using the gmx hbond command [61], using a maximum of 0.35 nm donor–acceptor distance and a maximum 30$^\circ $ hydrogen–donor–acceptor angle.

**Radius of gyration during MD.** Rg was computed using GROMACS [61] with default settings. For protein–aptamer complexes, two definitions were used: (i) the aptamer chain alone, and (ii) a combined protein–aptamer group. In each case, Rg values were evaluated over the full trajectory and reported as both time-series data and trajectory-averaged means.

**Root mean square deviation during MD.** RMSD was computed using the protein backbone for least-squares superposition and the full aptamer chain as the analysis group. All values were computed relative to the initial structure and reported as both time-series data and trajectory-averaged means.

#### Aptamer-only structures

Performance was evaluated using Rg and RMSD derived from MD simulations. RMSD was additionally computed in PyMOL [62], with each predicted aptamer aligned to the corresponding chain extracted from the experimental complex. This provides a consistent reference structure for comparison across methods, although the reference corresponds to the bound rather than the free-state conformation.

## Results

### Accuracy across protein–aptamer complexes

Our benchmark assesses the ability of current deep-learning models to predict protein–aptamer complexes, a class of interactions, i.e. not explicitly represented during model training. Complexes absent from the Boltz-2 training set are shown in red throughout the figures. Unless otherwise noted, analyses in this section are based on the top-ranked structures selected as described in Section Evaluation metrics.

**Figure 1:**
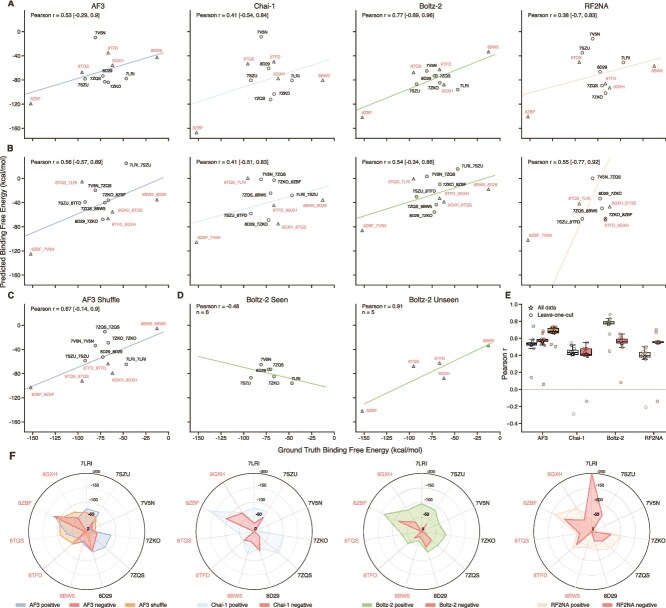
Correlation of $\Delta G_{\mathrm{bind}}$ values between ground truth and model-predicted structures. (A–C) Correlations between GT and positive predictions, mismatched negative predictions, and AF3 shuffled-sequence controls, respectively. The x-axis denotes $\Delta G_{\mathrm{bind}}$ from experimental structures, and the y-axis denotes the corresponding values from model-predicted structures. Negative labels follow the format protein_aptamer, indicating intentionally mismatched protein–aptamer pairs (e.g. 7LRI_7SZU indicates that the protein sequence is taken from 7LRI and the aptamer sequence from 7SZU). Shuffle labels denote cognate proteins paired with shuffled versions of the corresponding native aptamer sequences. Values in brackets denote the bootstrap confidence intervals for Pearson $r$. (D) Seen versus unseen split for Boltz-2 based on training-set inclusion. (E) LOO analysis of Pearson correlations after excluding each complex in turn. (F) Visualization of predicted $\Delta G_{\mathrm{bind}}$ values for the positive, mismatched negative, and AF3 shuffled-sequence sets, shown within the range 0 to −200 kcal mol$^{-1}$, with values above 0 truncated to 0 for clarity. 8BW5, 8TFD, 8TQS, 8ZBF and 9GXH were absent from the Boltz-2 training set. For RF2NA, the mismatched negative predictions for 7LRI_7SZU and 8BW5_8D29 yielded $\Delta G_{\mathrm{bind}}$ values of −214.07 and 598.95 kcal mol$^{-1}$, respectively. Both cases are excluded from panel (B) for clarity.

We evaluated the correspondence between experimental and predicted $\Delta G_{\mathrm{bind}}$ values for the positive and mismatched negative sets across the 11 benchmark complexes and all four models. AF3 shuffled-sequence controls were additionally included as a second negative-control strategy to reduce the possibility of non-specific binding in mismatched pairs. For the positive set, the models showed overall moderate correlation with the experimental values (Fig. 1A). Boltz-2 showed the highest Pearson correlation in the positive set, with six of the 11 benchmark complexes included in its training set. Across all models, the mismatched negative set showed Pearson correlations similar to those of the positive set and, in some cases, even closer agreement with the experimental values (Fig. 1B). These observations indicate that the models do not reliably distinguish native from mismatched protein–aptamer pairs, further indicating limited ability to capture aptamer specificity. This trend was even more pronounced for the AF3 shuffled-sequence controls: despite disruption of biologically meaningful aptamer sequence order, the shuffled controls yielded a Pearson correlation coefficient of $r=0.67$, which was higher than that of both the AF3 positive and mismatched negative sets (Fig. 1C). This pattern further suggests that AF3 does not reliably distinguish biologically meaningful aptamer sequences from nonbiological shuffled sequences and can still generate complexes with apparently reasonable binding affinities for such sequences.

To assess the stability of the MM/GBSA estimates within the selected trajectory window, we further compared $\Delta G_{\mathrm{bind}}$ across four consecutive 2.5 ns subwindows spanning 10.01–20.00 ns (Supplementary Fig. S1). The window-based MM/GBSA analysis showed clear system- and model-dependent variation. Some complexes exhibited relatively stable $\Delta G_{\mathrm{bind}}$ values across the four consecutive subwindows, whereas others showed more pronounced fluctuations, consistent with substantial system-dependent variation in temporal stability. In most cases, however, the 10.01–20.00 ns interval did not show a consistent directional drift, and the overall energy profiles remained within a broadly comparable range across subwindows. Overall, the analyzed trajectory window provides a reasonable basis for estimating $\Delta G_{\mathrm{bind}}$, although some complexes remain more sensitive to the choice of time window than others.

To assess whether the observed correlations were strongly influenced by individual complexes, we performed leave-one-out (LOO) analyses. Pearson correlations were recalculated after excluding each complex in turn. Across all models and control sets, the overall LOO distributions remained broadly consistent with the full-data correlations (Fig. 1E), indicating that the main correlation patterns were broadly preserved after omission of any single benchmark complex. At the same time, several outlying LOO values were observed for each model, highlighting the sensitivity of the correlations to individual complexes under this small-sample setting. Importantly, the main observations from the full-data analysis were preserved under LOO: Boltz-2 retained the highest positive-set correlations overall, the mismatched negative sets remained similar to the positive sets across models, and the AF3 shuffled-sequence controls continued to show comparably high correlations. The corresponding LOO Pearson correlation coefficients are provided in Supplementary Table S2.

We then performed bootstrap resampling (10 000 iterations) to estimate 95% confidence intervals for Pearson $r$ and thereby quantify uncertainty in the correlation estimates. Across the positive, mismatched negative, and AF3 shuffled-sequence sets, the bootstrap confidence intervals were generally broad, reflecting substantial sampling uncertainty in the correlation estimates (Fig. 1A–C; values in brackets denote the bootstrap confidence intervals). Accordingly, the $\Delta G_{\mathrm{bind}}$ correlations are more informative for identifying overall trends than for resolving subtle differences in Pearson correlation among models or control sets.

We next examined the potential effect of Boltz-2 training-set overlap by comparing its performance between benchmark complexes seen and unseen during training (Fig. 1D). The seen subset showed a negative Pearson correlation (−0.48), whereas the unseen subset showed a high positive correlation (0.91). This further highlights the instability of correlation estimates under such small subset sizes.

We further visualized $\Delta G_{\mathrm{bind}}$ patterns across the four models using radar charts (Fig. 1F). For Chai-1 and Boltz-2, the positive sets generally showed more favorable $\Delta G_{\mathrm{bind}}$ values than the mismatched negative sets across complexes. By contrast, AF3 showed broadly similar values across the positive, mismatched negative, and shuffled-sequence sets, without clear separation among the three groups. For RF2NA, the positive and mismatched negative sets likewise did not show clear separation, and the mismatched negative set exhibited similar or more favorable values for some complexes. These visual patterns are consistent with the correlation analyses and further show that favorable binding-free-energy estimates are not restricted to native protein–aptamer pairs. The corresponding $\Delta G_{\mathrm{bind}}$ values for the positive and negative sets are provided in Supplementary Tables S1.

We used PO as an atom-based measure to quantify the fraction of aptamer atoms remaining within the pocket region during MD simulations. PO profiles were generally stable over the 20 ns trajectories across complexes and models (Supplementary Fig. S2), indicating that the extent of pocket occupation usually remained at a relatively stable level throughout the analyzed interval. The correspondence between positive-set PO and GT was strongly complex-dependent. In some complexes, such as 7LRI and 8TQS, AF3, Chai-1, and Boltz-2 positive predictions showed PO values close to GT, indicating levels of pocket occupation during MD comparable with those of GT. In other cases, however, positive PO remained clearly below GT, such as AF3 for 7V5N and all models for 9GXH, suggesting lower retention of the predicted aptamer within the pocket region. Notably, mismatched negative controls across all models, and shuffled-sequence controls for AF3, also maintained high pocket occupation throughout the 20 ns MD simulations, and in some complexes even exhibited values comparable with or higher than those of the positive and GT sets. Together with the $\Delta G_{\mathrm{bind}}$ results, these data show that the models can generate non-native complexes showing both sustained pocket occupation and apparently favorable $\Delta G_{\mathrm{bind}}$. AF3 showed the same behavior even for biologically irrelevant shuffled sequences.

**Figure 2:**
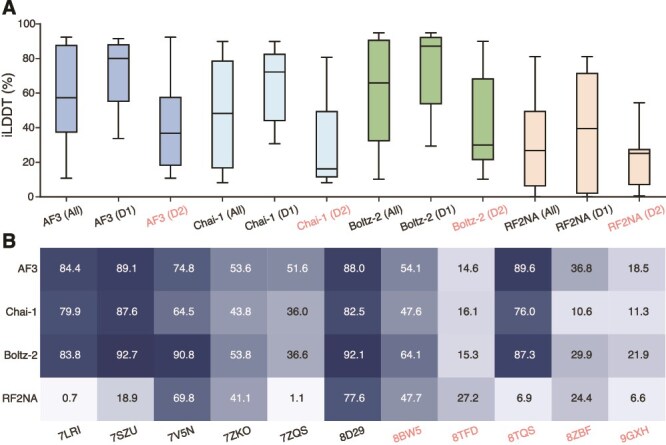
iLDDT scores across 25 predictions per complex. (A) All 11 protein–aptamer complexes (All), with subsets corresponding to complexes included (D1: 7LRI, 7SZU, 7V5N, 7ZKO, 7ZQS, and 8D29) or absent (D2: 8BW5, 8TFD, 8TQS, 8ZBF, and 9GXH) from the Boltz-2 training set. (B) Median iLDDT for each model on each complex.

Interface accuracy was assessed using iLDDT scores, which quantify the agreement of inter-chain contacts between the predicted and reference structures [39, 59]. Scores were computed for all 25 predictions per complex and reported on a 0%–100% scale, consistent with AF3. Thresholds of 23.6% and 77.6% correspond to the DockQ categories of “correct” and “very high accuracy,” respectively [39]. Across models, Boltz-2 achieved the highest overall iLDDT scores on the full benchmark (All, Fig. 2A), which may reflect training-set inclusion, as six benchmark complexes were present in its training data. AF3 ranked slightly lower, followed by Chai-1 and RF2NA. As six of the benchmark complexes are included in the Boltz-2 training set, we partitioned the dataset into D1 (the six training-set complexes) and D2 (the five unseen complexes). All models showed lower accuracy on D2 relative to D1, indicating that interface recovery was generally more challenging for complexes in the D2 subset.

In Fig. 2B, AF3 and Boltz-2 achieved high median iLDDT scores across most complexes, exceeding the “very high accuracy” threshold for 7LRI, 7SZU, 8D29, and 8TQS. In these cases, Boltz-2 performed comparably with or slightly better than AF3 (e.g. 90.8% in 7V5N versus 74.8% for AF3), consistent with the inclusion of these complexes in its training set. Performance decreased for all models on the remaining complexes. In 8TFD and 9GXH, AF3, Chai-1, and Boltz-2 failed to produce correct interfaces, whereas RF2NA succeeded in 8TFD but with relatively low accuracy (27.2%). Chai-1 generated correct predictions for several complexes and occasionally approached higher accuracy, but its overall performance remained below that of AF3 and Boltz-2. RF2NA produced correct interfaces for some complexes but achieved high accuracy less frequently. Five RF2NA predictions did not produce iLDDT scores, consistent with the absence of inter-chain contacts in these structures [59]. These cases were excluded from the analysis, resulting in fewer RF2NA data points relative to the other models. These results indicate clear system-dependent variation in interface recoverability across protein–aptamer complexes. For Boltz-2, the lower accuracy observed on D2 further suggests that highly accurate interface recovery remains more difficult for the subset of benchmark complexes not represented in its training set. The relationship between these interface-accuracy patterns and the model confidence metric interface predicted template modeling (ipTM)) is examined in the later section Interface confidences. The complete iLDDT values for all predictions are provided in Supplementary Table S3.

**Figure 3:**
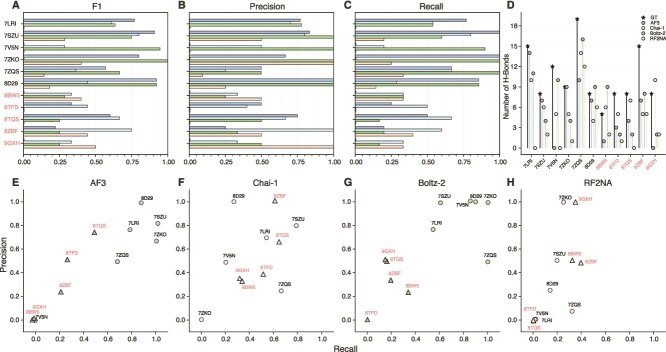
Interfacial H-bond analysis for protein–aptamer complexes. H-bonds were identified across 11 complexes, comparing GT with the top-ranked predictions from AF3 (blue), Chai-1 (cyan), Boltz-2 (green), and RF2NA (orange). (A–C) F1 score, precision, and recall. (D) Total number of H-bonds. (E–H) Precision-recall distributions for each model.

Interfacial H-bonds in the predicted complexes were evaluated by calculating precision, recall, and F1 scores against GT structures (Fig. 3A–C), revealing an overall trend consistent with the iLDDT analysis. F1 scores are shown in Fig. 3A and broadly followed the same trends as precision and recall. For recall (Fig. 3C), AF3 and Boltz-2 reproduced more than half of the native interfacial H-bonds in the six complexes included in the Boltz-2 training set, although several native H-bonds present in the GT structures were not captured in the predictions. A similar trend was observed for precision (Fig. 3B): approximately half of the predicted H-bonds matched native interfacial H-bonds, while additional non-native interactions were introduced. Chai-1 performed comparably with the other models, with slightly higher accuracy on the five complexes where AF3 and Boltz-2 showed reduced performance. RF2NA generally showed lower H-bond accuracy across complexes. RF2NA predictions include explicit hydrogen coordinates, unlike those of the other models, which could contribute to small discrepancies in H-bond counts. The combined precision and recall results indicate that, although all models were generally able to form interfacial H-bonds, their accuracy varied markedly across complexes, and they often failed to accurately reconstruct the native H-bond network while introducing additional non-native interactions. This variation is further illustrated in Fig. 3E–H, which show the precision–recall distributions for each model. AF3 and Boltz-2 achieved higher precision and recall for multiple complexes, whereas Chai-1 achieved comparable or slightly higher accuracy on complexes where AF3 and Boltz-2 performed less well. RF2NA remained consistently lower across all cases. Together, the lower precision, recall, and overall H-bond counts suggest that RF2NA is generally less effective at recovering interfacial organization consistent with the experimental structures. The total number of interfacial H-bonds per complex is shown in Fig. 3D. AF3, Chai-1, and Boltz-2 generated H-bond counts comparable to the GT for several complexes, whereas larger discrepancies were observed for others such as 8TFD and 8ZBF. RF2NA generally produced fewer interfacial H-bonds, consistent with its lower precision and recall. Importantly, agreement in the total number of interfacial H-bonds did not necessarily indicate accurate recovery of the native interface, as similar counts could still arise from different underlying contact patterns. A complete summary of H-bond counts is provided in Supplementary Table S4.

**Figure 4:**
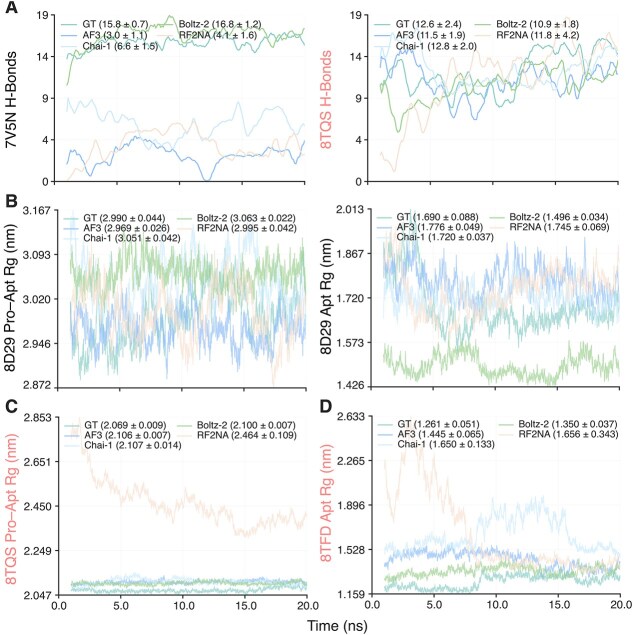
H-bonds and Rg profiles during 20 ns MD simulations. Trajectories are shown from 1 ns onward to exclude initial equilibration fluctuations. (A) Interfacial H-bond profiles for 7V5N and 8TQS. (B–D) Rg profiles for 8D29, 8TQS, and 8TFD. Values in parentheses indicate mean $\pm $ standard deviation over the trajectory.

To assess interfacial stability, we examined the temporal persistence of interfacial H-bonds over the analyzed MD interval. Unless otherwise noted, all MD-derived time-series profiles are shown from 1 ns onward to exclude the initial equilibration phase. The number and persistence of interfacial H-bonds showed clear complex- and model-dependent variation (Supplementary Fig. S3). In some cases, such as 7V5N (Fig. 4A), Boltz-2 reproduced H-bond levels close to the GT reference, whereas AF3, Chai-1, and RF2NA showed substantially lower values. In other systems, such as 8TQS (Fig. 4A), several models produced H-bond levels broadly comparable with the GT structure. Notably, mismatched negative controls and, for AF3, shuffled-sequence controls also maintained substantial numbers of interfacial H-bonds throughout the simulations (Supplementary Fig. S3). Although negative controls often showed fewer H-bonds than the corresponding positive predictions, this trend was not uniform, and in some complexes the negative or shuffled controls exhibited H-bond levels comparable with or higher than those of the positive set. These results indicate that even for non-native or shuffled protein–aptamer pairs, the predicted complexes can still maintain persistent interfacial interactions during MD, rather than dissociating rapidly during the initial phase of the simulation.

Structural stability was evaluated using Rg over the analyzed MD interval. Rg profiles showed clear complex- and model-dependent variation, indicating that the structural stability of the predicted complexes was not uniform across systems. For 8D29 and its aptamer component (Fig. 4B), the mean and standard deviation of Rg across models were close to the experimental reference, suggesting that the predicted structures remained stable under the present MD simulation conditions. In 8TQS (Fig. 4C), AF3, Chai-1, and Boltz-2 likewise produced Rg values close to the experimental reference, whereas RF2NA showed larger fluctuations, with a standard deviation of 0.109 nm. A similar pattern was observed for the 8TFD aptamer (Fig. 4D). Chai-1 and RF2NA showed greater variability (0.133 nm and 0.343 nm, respectively), indicating reduced structural stability. These results suggest that some predicted complexes can maintain overall compactness comparable with the experimental structures, whereas others remain more susceptible to structural fluctuations during MD. Rg profiles for all complexes are shown in Supplementary Figs S4–S5.

**Figure 5:**
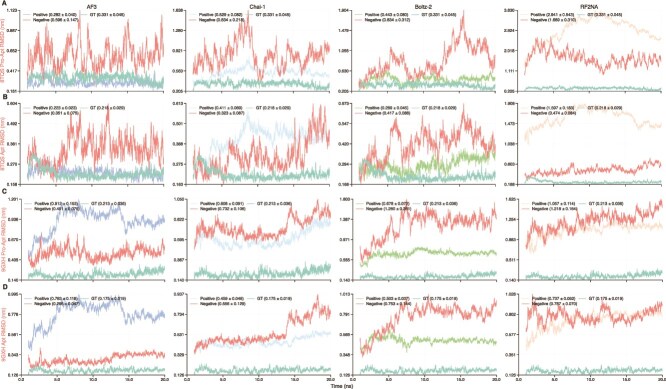
Aptamer RMSD during 20 ns MD simulations. (A, C) Aptamer RMSD relative to the protein backbone within the complex. (B, D) RMSD of the aptamer-only simulations. Curves show GT and positive predictions, with negative (mismatched) predictions in red. Values in parentheses indicate mean $\pm $ standard deviation over the trajectory.

We further evaluated structural deviations using both aptamer RMSD after protein-backbone alignment and aptamer-only RMSD. The RMSD profiles likewise showed clear complex- and model-dependent variation, consistent with the patterns observed for the other MD-derived metrics. In some systems, such as 8TQS (Fig. 5A and B), AF3 and Boltz-2 produced values close to the experimental reference in both analyses; by contrast, in 9GXH (Fig. 5C and D), all models showed RMSD values substantially higher than the experimental reference. To further dissect the origin of these structural deviations, we compared the aptamer-only RMSD with the aptamer RMSD after protein-backbone alignment. This comparison revealed two characteristic patterns. In some systems, the observed deviations were dominated by intrinsic aptamer conformational fluctuations, with only limited displacement relative to the protein. In others, the deviations involved not only conformational changes within the aptamer itself but also more pronounced displacement relative to the protein. The former pattern is illustrated by 8TQS, for which only small differences were observed between the two analyses across models and the experimental reference. The latter is exemplified by 9GXH, where all models showed clearly elevated RMSD values relative to the experimental reference in both analyses. Mismatched negative controls and, for AF3, shuffled-sequence controls exhibited larger deviations from the experimental reference than the corresponding positive predictions in many systems, but their RMSD profiles generally remained within a limited range rather than diverging rapidly. Taken together, these results indicate that model-predicted complexes can remain structurally stable over the simulation timescale while still deviating substantially from the native experimental interface. The RMSD profiles for both the protein-referenced and aptamer-only analyses are provided in Supplementary Figs S6 and S7.

**Figure 6:**
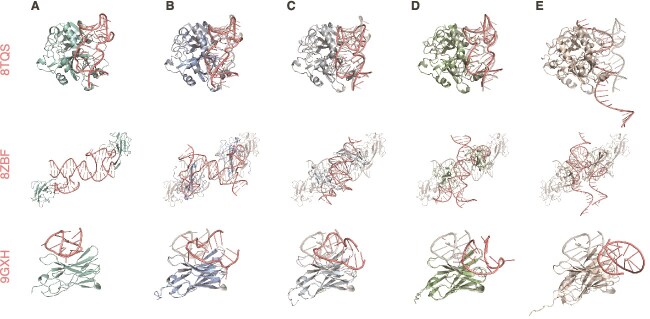
Representative protein–aptamer complexes. Shown are 8TQS, 8ZBF, and 9GXH. For each complex, (A) the experimental reference is compared with (B) AF3, (C) Chai-1, (D) Boltz-2, and (E) RF2NA.

Protein components were generally well captured by AF3, Chai-1, and Boltz-2, whereas RF2NA showed reduced accuracy. By contrast, aptamer conformations exhibited substantially larger deviations, indicating that aptamer modeling remains more challenging. For 8TQS (Fig. 6), AF3, Chai-1, and Boltz-2 produced complexes in close agreement with the experimental structure, particularly for the protein component. RF2NA, however, showed noticeable deviations in protein placement and did not fully recover the aptamer fold. In 9GXH, all models captured the overall protein arrangement consistent with the experimental reference, whereas the predominant variation arose from the aptamer. For more challenging systems such as 8ZBF, the predicted protein components showed substantial global translational or rotational shifts relative to the experimental reference, with the aptamer conformations likewise failing to align with the experimental fold, resulting in pronounced overall deviations. This pattern suggests that the major differences often arose from aptamer conformation and placement, although protein placement and structural inaccuracies also contributed in more challenging systems. All top-ranked predicted structures are provided in Supplementary Figs S8 and S9.

### Performance on aptamer-only structures

To assess whether the models can reconstruct aptamer conformations observed in the bound state once protein constraints are removed, we evaluated their predictions for aptamers in isolation. The GT reference in this section corresponds to the aptamer extracted from the experimental protein–aptamer complex and therefore reflects a bound-state reference rather than an isolated aptamer conformation.

**Figure 7:**
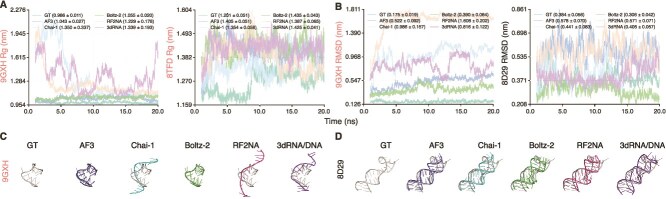
Rg and RMSD of aptamer-only structures during 20 ns MD simulations, along with representative aptamer-only structures. Aptamer sequences were extracted from experimental protein–aptamer complexes and used to generate aptamer-only predictions with five models. The GT reference corresponds to the aptamer component within the experimental complex, with Rg and RMSD values taken directly from the in-complex aptamer, serving as a bound-state reference rather than a representation of isolated aptamer behavior. Representative examples are shown: (A) Rg trajectories for 9GXH and 8TFD, (B) RMSD trajectories for 9GXH and 8D29, and (C,D) representative aptamer-only structures for 9GXH and 8D29, respectively.

Across all complexes, the aptamer Rg trajectories showed pronounced model- and complex-dependent variation, indicating that maintenance of compactness after removal of protein constraints varied substantially across methods (Supplementary Fig. S10). This pattern is illustrated by 9GXH and 8TFD (Fig. 7A). For 9GXH, AF3 and Boltz-2 produced Rg values close to the GT reference, whereas Chai-1 and RF2NA exhibited larger fluctuations before reaching a stable regime, and 3dRNA/DNA did not reach a comparably stable Rg regime over the simulation interval. By contrast, for 8TFD, all models produced Rg values broadly comparable with the GT reference. More broadly, AF3 and Boltz-2 more frequently remained close to the GT reference in Rg across targets, whereas RF2NA more often sampled expanded conformations and showed the largest deviations. Chai-1 and 3dRNA/DNA showed intermediate behavior but remained strongly target-dependent (Supplementary Fig. S10). These results indicate that some aptamer-only predictions can retain overall compactness consistent with the bound-state reference, although similarity in Rg alone does not guarantee recovery of the correct fold.

Consistent with the Rg analysis, RMSD also showed marked sequence- and model-dependent variation, but more directly reflected deviation from the bound-state reference geometry. For 9GXH (Fig. 7B), Chai-1, RF2NA, and 3dRNA/DNA exhibited substantially higher RMSD values than the GT reference, whereas AF3 and Boltz-2 remained closer to the GT trajectory. For 8D29 (Fig. 7B), most models produced RMSD values comparable with the reference, with only modest deviations across methods. Structural visualization further supported these differences. For 9GXH (Fig. 7C), the aptamers predicted by Chai-1, RF2NA, and 3dRNA/DNA did not adopt compact folds, consistent with their elevated RMSD values and indicating limited recovery of the bound-state fold in isolation. By contrast, for 8D29 (Fig. 7D), the predicted aptamers showed only minor RMSD fluctuations and retained folded conformations, indicating that some bound-state aptamer folds can be partially reconstructed even after removal of protein constraints. RMSD trajectories of all aptamer-only structures are provided in Supplementary Fig. S11, and the corresponding structural visualizations are shown in Supplementary Fig. S12. We additionally computed PyMOL-based RMSD values between the bound-state GT reference and the predicted structures, as reported in Supplementary Table S8.

### Interface confidences

ipTM and iLDDT were compared across all protein–aptamer complexes to assess whether confidence metrics reflect interface accuracy. The ipTM score is an AF3 confidence metric derived from the TM score [39, 63, 64], which quantifies the accuracy of predicted subunit arrangements within a complex. In AF3, confidence estimation is performed by a dedicated confidence model, i.e. trained in parallel with, but separately from, the structure prediction module, indicating that such scores are best interpreted as model-internal estimates of prediction reliability rather than direct measures of experimental correctness [39]. ipTM values above 0.8 correspond to high-quality predictions, whereas values below 0.6 indicate incorrect assemblies; intermediate values (0.6–0.8) reflect uncertain prediction quality [39].

**Figure 8:**
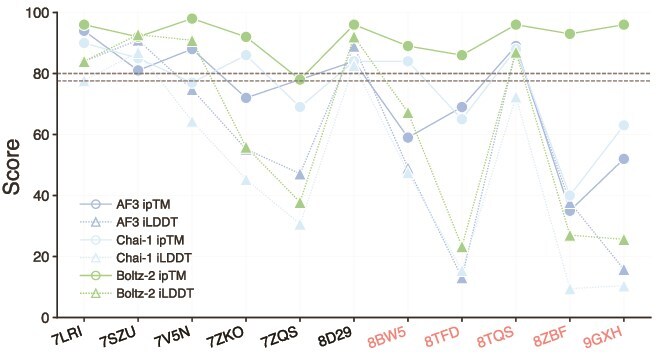
ipTM and iLDDT scores for predicted protein–aptamer structures. Circles denote ipTM and triangles denote iLDDT. Dashed lines indicate thresholds for high-quality predictions on the rescaled 0–100 axis: 80 for ipTM and 77.6 for iLDDT.

For visualization, ipTM and iLDDT values were scaled to a 0–100 range. As shown in Fig. 8, the relationship between ipTM and iLDDT varied substantially across models. Boltz-2 assigned uniformly high ipTM values (>80) across nearly all complexes, indicating consistently high internal confidence in its interface predictions. However, several complexes, such as 8TFD, 8ZBF, and 9GXH, exhibited markedly lower iLDDT values, indicating reduced interface accuracy despite the high ipTM confidence. In contrast, AF3 and Chai-1 showed greater consistency between ipTM and iLDDT across complexes. Confidence metrics, including pTM, ipTM, internal confidence scores, and iLDDT, are provided in Supplementary Table S9.

Our results indicate that a high confidence score does not necessarily guarantee high interface accuracy in protein–aptamer systems. In particular, Boltz-2 produced uniformly high confidence scores across nearly all complexes, whereas the corresponding iLDDT values remained substantially more variable. This pattern is consistent with overconfidence, in which the model assigns high internal confidence even when the predicted interface deviates substantially from the experimental structure. It remains unclear whether this reflects a broader property of the model or a behavior, i.e. particularly pronounced in protein–aptamer systems.

One possible explanation is that the confidence module of Boltz-2, although similar to that of AF3, is not identical. Its confidence module uses a deeper PairFormer stack and separates the PDE/PAE prediction heads for intra-chain and inter-chain pairs, indicating that its representation of interface error and confidence is distinct from that of AF3. Boltz-2 also incorporates ensemble supervision, B-factor supervision and a modified MSA sampling strategy during structure/confidence training. Consequently, the model is trained not only on single static conformations, but also on local fluctuations, structural heterogeneity and dynamic features derived from experimental ensembles and MD trajectories. Such a training regime may broaden the range of conformations that the model considers plausible, thereby leading to relatively high internal confidence even when predictions are structurally divergent. The training-data mixture of Boltz-2 also differs from that of AF3, with a relatively high proportion of MD-derived data alongside multiple complex-distillation datasets. Although Boltz-2 includes an affinity module, this is unlikely to directly account for the ipTM overconfidence observed in this study, as affinity training is performed only after structure and confidence training with gradients detached from the trunk. Notably, ipTM is also used within the affinity pipeline for structure selection and sample filtering. If ipTM in small-molecule systems likewise shows systematic overestimation, such bias could in principle propagate to downstream affinity-related predictions. Collectively, these differences in confidence-module design, training strategy, and data composition may contribute to the uniformly elevated ipTM values observed in this study.

Therefore, ipTM should be interpreted with caution, particularly for Boltz-2 and, more broadly, for protein–nucleic acid systems, including protein–aptamer complexes. Given that previous work has shown ipTM to be strongly correlated with DockQ [39], we suggest that, in such systems, ipTM should be evaluated together with independent interface-quality metrics such as iLDDT rather than interpreted alone or assessed solely through DockQ.

### Effect of ions on protein–aptamer complexes

Metal ions play essential roles in nucleic acid folding and stabilization, particularly for aptamers whose conformations often depend on mono- and divalent cations, such as Mg^2+^, K^+^, and Ca^2+^ [65–67]. To assess the impact of metal-ion inclusion on model predictions, we evaluated protein–aptamer complexes containing experimentally resolved metal ions using the same benchmark set, excluding 7V5N and 8TFD, which lack metal ions in their experimental structures. For each complex, ions were included together with the protein and aptamer sequences in the model input, according to the ion type and stoichiometry observed in the corresponding GT structure. For example, if two Mg^2+^ ions were present in the experimental structure, two corresponding ion entries were supplied. In AF3 and Boltz-2, ions were represented as separate ligand entries using the appropriate PDB Chemical Component Dictionary (CCD) codes (e.g. CA for calcium), whereas in Chai-1 they were provided as ligand chemical strings (e.g. [Ca+2]). By contrast, RF2NA does not provide an explicit ion input entry in its current input pipeline; therefore, ion-inclusion analysis was performed only for AF3, Chai-1, and Boltz-2. The effect of ion inclusion on interface accuracy and binding free energy was then quantified using iLDDT and $\Delta G_{\mathrm{bind}}$ and compared with the corresponding ion-free results.

No significant differences in iLDDT were observed between ion-free and ion-included predictions for AF3 and Chai-1 across the nine ion-containing complexes (25 predictions per complex), whereas Boltz-2 exhibited a small but statistically significant improvement. These results suggest that explicit ion input had at most a limited and model-dependent effect on interface accuracy in this analysis. In some cases, such as 8TQS and 8ZBF, Chai-1 did not incorporate the corresponding Mg^2+^ and Na^+^ ions into its predicted structures. PyMOL visualizations further showed that, even when ions were included, their placement did not always match the GT arrangement; in several complexes, ions located on the aptamer side in the GT structures were instead placed on the protein side or redistributed differently in the predictions (Supplementary Figs S13 and S14), suggesting that ion-associated features remained difficult to capture accurately and that correct ion placement was not consistently achieved in the predicted complexes. Detailed statistics and full iLDDT results are provided in Supplementary Tables S5 and S6.

**Figure 9:**
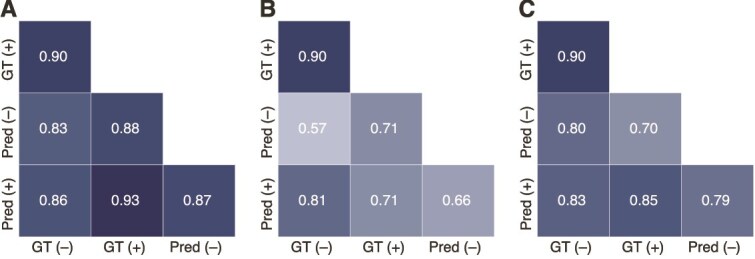
Cross-correlations of $\Delta G_{\mathrm{bind}}$ across nine protein–aptamer complexes. (A–C) show AF3, Chai-1, and Boltz-2, respectively. “+” and “–” indicate with or without ions, respectively. “Pred” denotes predicted structures (e.g. “Pred +” refers to predictions with ions). Pearson correlation coefficients ($r$) of $\Delta G_{\mathrm{bind}}$ values are shown for each model.

We further assessed the cross-correlation of $\Delta G_{\mathrm{bind}}$ values derived from ion-included and ion-free predictions. $\Delta G_{\mathrm{bind}}$ was computed from the top-ranked predictions following the procedure described in Section Experimental setup. The results indicate that overall trends in $\Delta G_{\mathrm{bind}}$ were largely consistent across ion conditions. Across nine complexes, ion-included (Pred +) and ion-free (Pred−) predictions from all models showed positive correlations in $\Delta G_{\mathrm{bind}}$ (Fig. 9). Correlations between the predictions and their corresponding experimental references (Pred$+$ and GT$+$; Pred− and GT−) exhibited similar patterns, indicating that ion inclusion did not markedly alter the overall energetic trends. Given the limited sample size, this consistency reflects general trend alignment rather than statistically resolved precision. GT$+$ and GT− remained strongly correlated because their protein and aptamer coordinates are identical, differing only in the presence of ion coordinates in the PDB files. Correlations between Pred− and GT− across the nine complexes were higher than those based on all 11 complexes (Fig. 1), indicating that the inclusion or exclusion of individual complexes can substantially influence the overall correlation under a limited sample size. All $\Delta G_{\mathrm{bind}}$ values for ion-related analyses are provided in Supplementary Table S7.

## Discussion

Predictions generated without ions showed clear limitations in both structural and interfacial accuracy. Consistent with the stereochemical and structural limitations reported for AF3 [39], the evaluated models showed limited ability to recover correct protein conformations and aptamer folds in large homomeric systems. In these cases, chain misalignment and structural deviations were often observed in the aptamer region, as exemplified by 7ZQS and 8ZBF. Additional deviations were observed in binding orientation, including reversed aptamer placement or inaccurate interfaces, as observed for 8BW5 and 8TFD. This may partly reflect the relatively limited amount of protein–nucleic acid structural data, especially for protein–aptamer complexes, available for training, as well as the greater conformational flexibility of aptamers and their more variable binding poses. Despite these inaccuracies, many predicted structures remained physically reasonable over the analyzed timescale, retaining overall conformations and a subset of native interfacial contacts during MD.

A key finding of this study is that current models have limited ability to distinguish native from non-native protein–aptamer pairs. This was evident in the $\Delta G_{\mathrm{bind}}$ analysis, in which positive and mismatched negative sets frequently showed overlapping or similarly correlated energetic patterns across systems. This interpretation was further supported by shuffled-sequence controls and PO analysis. In multiple systems, mismatched negative pairs and, for AF3, even nonbiological shuffled sequences yielded substantial pocket occupation, persistent interfacial H-bonds, and apparently favorable $\Delta G_{\mathrm{bind}}$ values. RMSD analyses further showed that such complexes often remained structurally stable over the simulation timescale while still deviating substantially from the native experimental interface. Taken together, these findings suggest that the limitations of current frameworks extend beyond structural inaccuracies alone. They may also involve a tendency to generate superficially plausible bound-like complexes from paired inputs, even when such complexes do not reflect biologically meaningful aptamer specificity or molecular recognition. At least a subset of these predictions may reflect nonspecific surface adherence rather than true native recognition. These findings underscore the importance of benchmark designs that explicitly test specificity rather than infer it indirectly from a single structural or energetic metric. The underlying causes of this tendency remain to be clarified.

Including ions in the model inputs produced no significant improvement in interface accuracy, and predicted ion positions were not always consistent with the GT arrangement. In some cases, ions were misplaced to the opposite side of the protein or aptamer, whereas in others they were positioned abnormally close to one another or clustered together. These observations point to persistent inaccuracies in ion placement across models. In some systems, particularly those involving G-quadruplex or duplex/G-quadruplex folds, the overall fold could still appear similar to the GT despite an incorrect ion configuration, suggesting that the models may reproduce the overall geometry without accurately representing ion-supported aptamer folding. Only marginal energetic differences in isolated cases. Ion inclusion did not materially alter the overall pattern of $\Delta G_{\mathrm{bind}}$, and consistent trends were observed under both ion-included and ion-free conditions. These findings indicate that ion effects have minimal influence on the overall protein–aptamer modeling accuracy within the examined setting. In addition, predicted ion positions often deviated from the experimental coordinates, indicating persistent limitations in geometric accuracy at ion-binding sites. It should also be noted that, under the current MD setting, only NaCl was used for system neutralization, and the ion-specific structural environment of each aptamer was not incorporated. The distinct stabilizing roles of ions such as Mg$^{2+}$, K$^{+}$, or Ca$^{2+}$ were therefore not explicitly modeled in individual systems. This limitation may be particularly relevant for ion-dependent folds, including G-quadruplex and related structures such as the G-quadruplex systems 7ZKO and 9GXH and the duplex/G-quadruplex DNA system 8BW5. The aptamer structural classes and experimentally resolved ions of all benchmark complexes are summarized in Supplementary Table S11.

For aptamers predicted in isolation, model performance remained mixed. Some systems adopted stable folds resembling the experimental references, whereas others showed larger conformational fluctuations and reduced stability, indicating that maintaining correct aptamer conformations across sequences remains challenging. For 3dRNA/DNA, DNA secondary structures were generated using the RNA prediction setting, which may have contributed to reduced DNA modeling accuracy. Future work may benefit from DNA-specific secondary-structure prediction tools, such as X3DNA-DSSR [68], to provide more appropriate inputs and potentially improve DNA structure modeling.

Although longer MD simulations can provide a more complete view of conformational adaptation, the benchmark used here requires simulations across a large number of predicted complexes and control sets, making substantially longer trajectories computationally demanding. Our 20 ns simulations were sufficient to reveal clear differences in structural stability, interface maintenance, and pocket occupation across models, even though some quantitative descriptors remain sensitive to longer timescales. Our additional 100 ns MD simulations suggest that the effect of simulation length depends on both the metric and the complex studied. The influence was greater for energetic estimates and fine-scale interfacial descriptors than for coarse-grained pocket association (Supplementary Fig. S15). In particular, $\Delta G_{\mathrm{bind}}$ and interfacial H-bond counts varied across consecutive 20 ns subwindows (Supplementary Fig. S15C and E), indicating that their quantitative values may remain sensitive to the analysis window over longer trajectories. Rg and RMSD likewise exhibited system-dependent time evolution (Supplementary Fig. S15A and B). Although the RMSD of the aptamer itself remained comparatively stable, the aptamer RMSD after protein-backbone alignment continued to increase beyond 20 ns in some complexes, suggesting that short simulations may not fully capture slower conformational adjustments in all cases. By contrast, PO remained comparatively stable across the 20–100 ns interval (Supplementary Fig. S15D), indicating lower sensitivity to simulation length.

A broader limitation of the field remains the scarcity of experimentally resolved protein–aptamer complexes. This constrains both the diversity of possible training data and the breadth of benchmarking. As structural databases expand, future benchmarks should incorporate newly resolved complexes, more explicit specificity-oriented controls, and complementary interface-aware metrics. In addition to salt bridges, residue-wise RMSD, and other MD-based descriptors, future evaluations could benefit from metrics targeting nucleic-acid-specific features such as base-pairing and base-stacking accuracy, as suggested by Ochoa *et al*. [49]. Such metrics could improve interpretability and enable more systematic cross-model comparisons.

Accurate prediction of protein–aptamer interactions remains a substantial challenge, reflecting both the intrinsic complexity of molecular recognition and the distinctive structural properties of aptamer. Beyond direct comparisons among models, the present study highlights the importance of establishing standardized, multidimensional, and specificity-aware benchmark designs for protein–aptamer modeling. Our results indicate that progress in the field should not be assessed solely on the basis of structural plausibility, energetic agreement, or the ability to generate stable bound-like complexes. It should instead be judged by whether models can reliably recover biologically meaningful, sequence-specific, and interface-specific recognition. From a broader perspective, the benchmark framework established here provides a systematic basis for evaluating predicted structures in protein–aptamer systems and may also inform more general assessment of protein–nucleic acid modeling.

**Key points**

- We establish a multidimensional benchmark for protein–aptamer complexes that integrates interface accuracy, molecular dynamics (MD)-derived stability metrics, pocket occupancy (PO), interfacial hydrogen-bond analysis, and energetic evaluation, and that may also inform benchmarking of broader protein-nucleic acid systems.
- Current artificial intelligence models show limited ability to distinguish native from non-native protein–aptamer pairs, as mismatched and shuffled controls can still yield favorable binding energies, sustained PO, and persistent interfacial contacts.
- Structural plausibility and MD stability alone are insufficient to assess prediction quality in protein–aptamer systems; specificity-aware benchmarking is required to evaluate biologically meaningful recognition.

## Supplementary Material

BIB_Jiani_supp_final_bbag206

## Data Availability

All experimental structures used in this study were obtained from the PDB and are publicly accessible. We used PDB entries with the following accession codes: 7LRI, 7SZU, 7V5N, 7ZKO, 7ZQS, 8D29, 8BW5, 8TFD, 8TQS, 8ZBF, and 9GXH. Processed structure files and selected analysis scripts can be accessed via GitHub (https://github.com/Brock-Biomedical-Data-Science-Lab/aptamer-benchmark).
