# Supplementary material for: Comprehensive evaluation of artificial intelligence-empowered approaches for protein–aptamer complex prediction
Source: Brief Bioinform. 2026 May 4;27(3):bbag206. doi: 10.1093/bib/bbag206 (PMC13137337; doi:10.1093/bib/bbag206)
Supplement: BIB_Jiani_supp_final_bbag206 [file bib_jiani_supp_final_bbag206.pdf]

Briefings in Bioinformatics, 2025, pp. 1–32

doi: DOI HERE

Advance Access Publication Date: Day Month Year

Paper

PAPER

Supplementary File for "Comprehensive Evaluation of Artificial Intelligence-Empowered Approaches for Protein-Aptamer Complex Prediction"

Jiani Zhao,[1] Kha Tram,[2] Hongbin Yan[3] and Yifeng Li[1,4*]

[1]Department of Computer Science, Brock University, 1812 Sir Isaac Brock Way, St. Catharines, L2S 3A1, Ontario, Canada, [2]Cytodiagnostics Inc., 919 Fraser Dr Unit 11, Burlington, L7L 4X8, Ontario, Canada, [3]Department of Chemistry, Department of Biological Sciences, Brock University, 1812 Sir Isaac Brock Way, St. Catharines, L2S 3A1, Ontario, Canada and [4]Department of Biological Sciences, Brock University, 1812 Sir Isaac Brock Way, St. Catharines, L2S 3A1, Ontario, Canada

*Corresponding author. yli2@brocku.ca

FOR PUBLISHER ONLY Received on Date Month Year; revised on Date Month Year; accepted on Date Month Year

Abstract

This document contains all the supplementary tables and figures mentioned in the main text.

© The Author 2025. Published by Oxford University Press. All rights reserved. For permissions, please e-mail: journals.permissions@oup.com

Table S1. Binding free energies (ΔG_{bind}) of the top-ranked predictions for protein-aptamer complexes without ions. Values are reported in kcal mol^{-1}. Negative labels follow the format protein_aptamer, indicating intentionally mismatched proteins-aptamers pairs (e.g., 7LRI_7SZU indicates that the protein sequence is taken from 7LRI and the aptamer sequence from 7SZU). Shuffle labels denote cognate proteins paired with shuffled versions of the corresponding native aptamer sequences. GT values apply only to the positive set, and shuffle-sequence results were calculated only for AF3. Complex names in red indicate cases absent from the Boltz-2 training set.

Set	Complex	AF3	Chai-1	Boltz-2	RF2NA	GT
Positive	7lri	-77.64	-80.80	-95.94	-51.03	-46.71
	7szu	-78.10	-80.63	-86.98	-34.97	-92.12
	7v5n	-9.78	-8.54	-65.06	-11.76	-80.76
	7zko	-83.97	-103.31	-84.77	-101.73	-66.45
	7zqs	-82.93	-112.34	-73.04	-89.34	-70.38
	8d29	-73.32	-60.84	-73.20	-66.38	-72.52
	8bw5	-42.55	-80.63	-33.25	-56.80	-12.76
	8tfd	-35.00	-49.65	-62.54	-86.01	-66.70
	8tqs	-67.11	-52.97	-67.58	-50.42	-95.47
	8zbf	-118.93	-166.95	-141.65	-140.46	-152.11
	9gxh	-55.54	-77.53	-87.49	-92.80	-61.94
Negative	7lri_7szu	25.16	-27.90	15.65	-214.07	–
	7szu_8tfd	-38.89	-58.07	-30.22	-66.96	–
	7v5n_7zqs	-19.62	-1.70	3.39	0.26	–
	7zko_8zbf	-35.91	-2.98	-9.57	-66.30	–
	7zqs_8bw5	-40.15	-24.45	-43.45	-49.51	–
	8d29_7zko	-67.75	-68.88	-55.69	-33.40	–
	8bw5_8d29	-35.14	-35.73	-17.95	598.95	–
	8tfd_9gxh	-66.24	-44.49	-32.68	-67.93	–
	8tqs_7lri	-5.41	0.43	-0.39	-41.70	–
	8zbf_7v5n	-124.88	-105.51	-85.91	-101.87	–
	9gxh_8tqs	-55.23	-74.94	-38.71	-46.76	–
Shuffle	7lri_7lri	-64.70	–	–	–	–
	7szu_7szu	-58.57	–	–	–	–
	7v5n_7v5n	-33.64	–	–	–	–
	7zko_7zko	-29.02	–	–	–	–
	7zqs_7zqs	-10.62	–	–	–	–
	8d29_8d29	-52.54	–	–	–	–
	8bw5_8bw5	-5.15	–	–	–	–
	8tfd_8tfd	-63.42	–	–	–	–
	8tqs_8tqs	-91.99	–	–	–	–
	8zbf_8zbf	-102.83	–	–	–	–
	9gxh_9gxh	-79.09	–	–	–	–

Table S2. Leave-one-out (LOO) Pearson correlation analysis of ΔG_{bind}. For each removed complex, the table reports the recalculated LOO Pearson correlation coefficient, followed in parentheses by the change relative to the corresponding full-dataset Pearson correlation coefficient (e.g., +0.06 indicates that the Pearson correlation increased by 0.06 after removing that complex). Negative labels denote intentionally mismatched protein-aptamer pairs, and Shuffle labels denote cognate proteins paired with shuffled versions of their corresponding native aptamer sequences. Shuffle-sequence results were calculated only for AF3. Complex names in red indicate cases absent from the Boltz-2 training set.

Set	Removed complex	AF3	Chai-1	Boltz-2	RF2NA
Positive	7lri	0.59 (+0.06)	0.43 (+0.02)	0.88 (+0.11)	0.35 (-0.03)
	7szu	0.52 (-0.01)	0.42 (+0.00)	0.77 (+0.00)	0.48 (+0.09)
	7v5n	0.74 (+0.21)	0.55 (+0.14)	0.80 (+0.02)	0.50 (+0.12)
	7zko	0.56 (+0.03)	0.44 (+0.02)	0.78 (+0.01)	0.43 (+0.04)
	7zqs	0.54 (+0.02)	0.44 (+0.03)	0.77 (+0.00)	0.40 (+0.01)
	8d29	0.53 (+0.00)	0.41 (+0.00)	0.77 (+0.00)	0.38 (+0.00)
	8bw5	0.48 (-0.05)	0.52 (+0.11)	0.65 (-0.12)	0.38 (+0.00)
	8tfd	0.54 (+0.01)	0.41 (+0.00)	0.77 (+0.00)	0.40 (+0.02)
	8tqs	0.54 (+0.01)	0.48 (+0.07)	0.83 (+0.06)	0.44 (+0.06)
	8zbf	0.14 (-0.39)	-0.29 (-0.70)	0.45 (-0.32)	-0.21 (-0.59)
	9gxh	0.52 (-0.01)	0.41 (+0.00)	0.79 (+0.02)	0.42 (+0.04)
Negative	7lri_7szu	0.52 (-0.04)	0.39 (-0.02)	0.49 (-0.05)	0.70 (+0.15)
	7szu_8tfd	0.58 (+0.01)	0.39 (-0.02)	0.55 (+0.00)	0.54 (-0.01)
	7v5n_7zqs	0.59 (+0.02)	0.47 (+0.06)	0.60 (+0.06)	0.55 (+0.00)
	7zko_8zbf	0.56 (+0.00)	0.41 (+0.00)	0.54 (+0.00)	0.56 (+0.01)
	7zqs_8bw5	0.56 (+0.00)	0.41 (+0.00)	0.56 (+0.02)	0.55 (+0.00)
	8d29_7zko	0.58 (+0.02)	0.43 (+0.02)	0.58 (+0.04)	0.55 (+0.00)
	8bw5_8d29	0.66 (+0.10)	0.48 (+0.07)	0.61 (+0.06)	-0.14 (-0.68)
	8tfd_9gxh	0.59 (+0.03)	0.41 (+0.00)	0.55 (+0.01)	0.56 (+0.01)
	8tqs_7lri	0.68 (+0.11)	0.55 (+0.14)	0.65 (+0.10)	0.55 (+0.00)
	8zbf_7v5n	0.06 (-0.51)	-0.14 (-0.55)	0.08 (-0.46)	0.67 (+0.12)
	9gxh_8tqs	0.59 (+0.02)	0.48 (+0.07)	0.57 (+0.03)	0.56 (+0.01)
Shuffle	7lri_7lri	0.73 (+0.06)	–	–	–
	7szu_7szu	0.67 (+0.00)	–	–	–
	7v5n_7v5n	0.70 (+0.03)	–	–	–
	7zko_7zko	0.68 (+0.01)	–	–	–
	7zqs_7zqs	0.73 (+0.06)	–	–	–
	8d29_8d29	0.67 (+0.00)	–	–	–
	8bw5_8bw5	0.53 (-0.14)	–	–	–
	8tfd_8tfd	0.68 (+0.01)	–	–	–
	8tqs_8tqs	0.66 (-0.01)	–	–	–
	8zbf_8zbf	0.50 (-0.17)	–	–	–
	9gxh_9gxh	0.73 (+0.06)	–	–	–

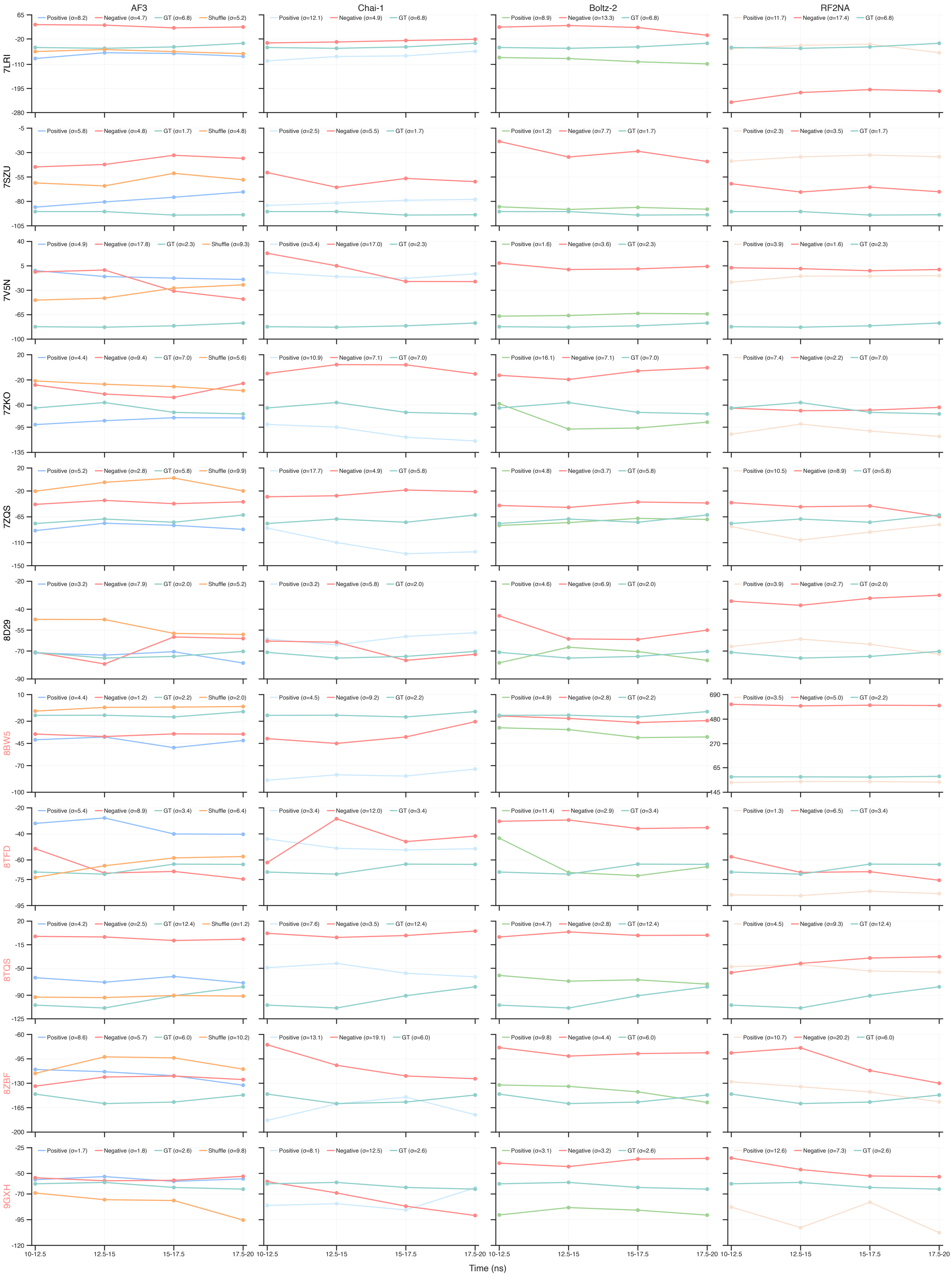

Fig. S1. Window-based ΔG_{bind} profiles across four consecutive 2.5 ns sub-windows (10.01–20.00 ns). Positive (native sequence pairs), Negative (intentionally mismatched pairs), and Shuffle (cognate proteins paired with shuffled native aptamers) are shown; Shuffle was calculated only for AF3. Values in parentheses denote standard deviation. Complex names in red indicate cases absent from the Boltz-2 training set.

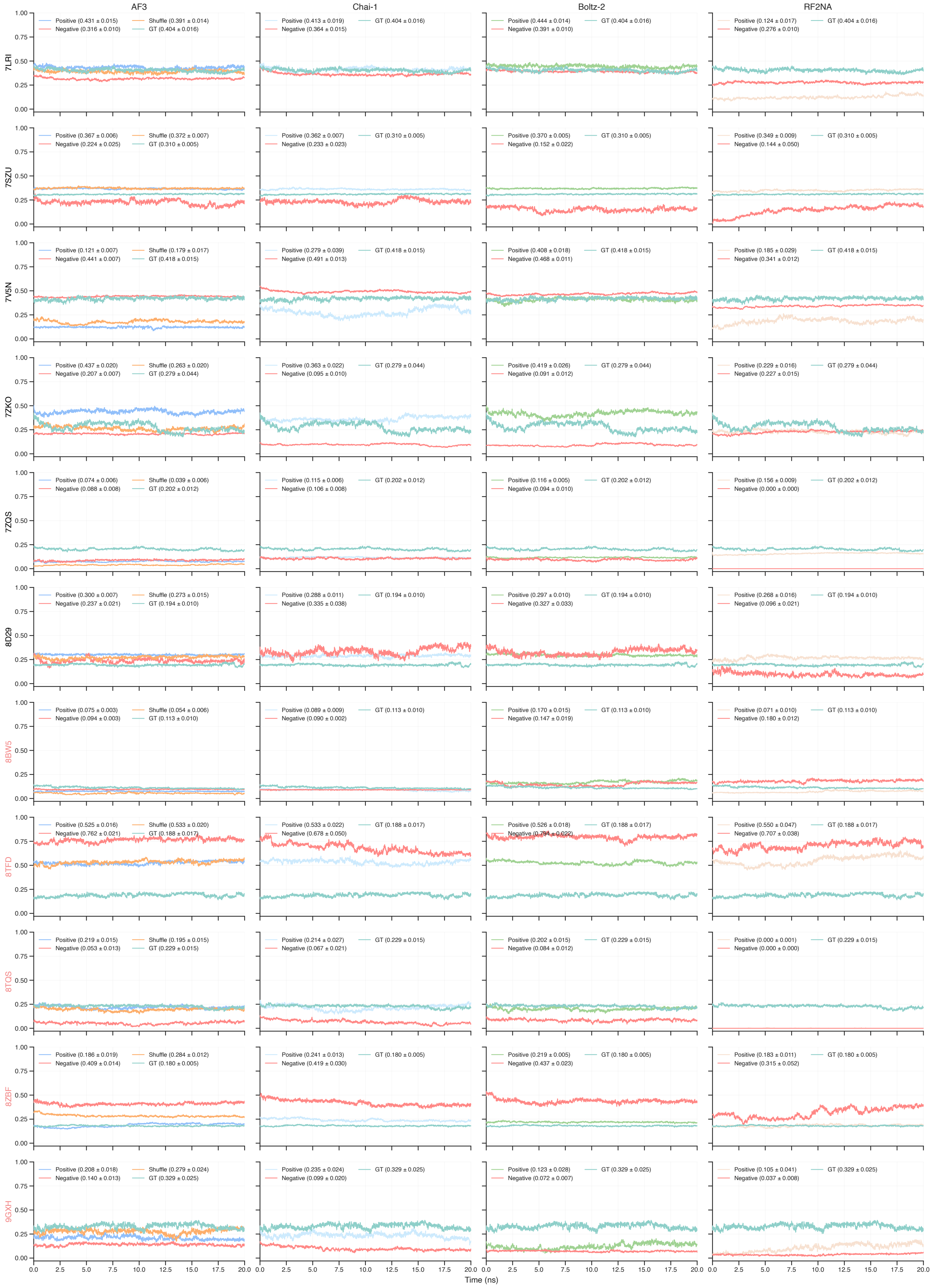

Fig. S2. Pocket occupancy (PO) profiles during MD simulations. PO was calculated as the fraction of total aptamer atoms that remained in contact with the binding pocket over time. Positive denotes native protein-aptamer pairs, Negative denotes intentionally mismatched pairs, and Shuffle denotes cognate proteins paired with shuffled native aptamer sequences; Shuffle was calculated only for AF3. Values in parentheses denote mean ± standard deviation. Complex names in red indicate cases absent from the Boltz-2 training set.

Table S3. iLDDT values for 25 predictions per complex. AF3, Chai-1, and Boltz-2 were each run with five seeds and five samples per seed, yielding 25 predictions per complex. RF2NA does not use diffusion sampling; its 25 predictions are labeled in the `ID` column for consistency. Complex names in red indicate cases absent from the Boltz-2 training set.

Complex	ID	AF3	Chai-1	Boltz-2	RF2NA
7lri	seed1_sample0	0.839	0.805	0.839	0.008
	seed1_sample1	0.819	0.810	0.839	0.005
	seed1_sample2	0.839	0.791	0.837	0.027
	seed1_sample3	0.845	0.798	0.834	0.026
	seed1_sample4	0.846	0.814	0.840	0.001
	seed2_sample0	0.844	0.799	0.840	0.003
	seed2_sample1	0.844	0.812	0.836	0.007
	seed2_sample2	0.840	0.777	0.836	0.006
	seed2_sample3	0.841	0.790	0.838	0.003
	seed2_sample4	0.842	0.800	0.837	0.005
	seed3_sample0	0.845	0.784	0.840	0.023
	seed3_sample1	0.843	0.799	0.845	0.006
	seed3_sample2	0.836	0.796	0.834	0.007
	seed3_sample3	0.848	0.786	0.842	0.002
	seed3_sample4	0.844	0.807	0.835	0.017
	seed4_sample0	0.842	0.790	0.835	0.002
	seed4_sample1	0.840	0.801	0.839	0.009
	seed4_sample2	0.845	0.778	0.837	0.005
	seed4_sample3	0.847	0.805	0.843	0.003
	seed4_sample4	0.845	0.782	0.839	0.012
	seed5_sample0	0.842	0.782	0.837	0.006
	seed5_sample1	0.846	0.802	0.838	0.025
	seed5_sample2	0.844	0.816	0.836	0.009
	seed5_sample3	0.840	0.787	0.837	0.021
	seed5_sample4	0.846	0.801	0.841	0.024
7szu	seed1_sample0	0.891	0.879	0.925	0.111
	seed1_sample1	0.889	0.885	0.923	0.775
	seed1_sample2	0.893	0.874	0.932	0.795
	seed1_sample3	0.906	0.876	0.925	0.112
	seed1_sample4	0.908	0.888	0.927	0.136
	seed2_sample0	0.908	0.866	0.936	0.146
	seed2_sample1	0.894	0.886	0.928	0.773
	seed2_sample2	0.888	0.881	0.929	0.125
	seed2_sample3	0.878	0.881	0.948	0.178
	seed2_sample4	0.890	0.881	0.928	0.801
	seed3_sample0	0.905	0.887	0.926	0.802
	seed3_sample1	0.907	0.864	0.925	0.782
	seed3_sample2	0.884	0.876	0.925	0.145
	seed3_sample3	0.881	0.880	0.930	0.161
	seed3_sample4	0.885	0.874	0.923	0.123
	seed4_sample0	0.898	0.878	0.928	0.810
	seed4_sample1	0.914	0.869	0.941	0.783
	seed4_sample2	0.882	0.875	0.923	0.189
	seed4_sample3	0.903	0.872	0.932	0.186
	seed4_sample4	0.891	0.898	0.921	0.765
	seed5_sample0	0.877	0.895	0.927	0.800
	seed5_sample1	0.884	0.870	0.917	0.084
	seed5_sample2	0.909	0.875	0.929	0.808
	seed5_sample3	0.883	0.871	0.922	0.332
	seed5_sample4	0.909	0.863	0.918	0.187
7v5n	seed1_sample0	0.747	0.615	0.898	0.714
	seed1_sample1	0.729	0.644	0.929	0.704
	seed1_sample2	0.768	0.695	0.906	0.705
	seed1_sample3	0.781	0.627	0.905	0.701
	seed1_sample4	0.778	0.604	0.905	0.687
	seed2_sample0	0.748	0.649	0.909	0.711
	seed2_sample1	0.777	0.622	0.911	0.077
	seed2_sample2	0.779	0.709	0.899	0.712
	seed2_sample3	0.700	0.675	0.904	0.692
	seed2_sample4	0.752	0.613	0.908	0.707
	seed3_sample0	0.772	0.571	0.935	0.015
	seed3_sample1	0.768	0.695	0.922	0.694
	seed3_sample2	0.760	0.675	0.897	0.669
	seed3_sample3	0.738	0.633	0.899	0.710

	seed3_sample4	0.704	0.657	0.905	0.698
	seed4_sample0	0.772	0.652	0.922	0.705
	seed4_sample1	0.752	0.650	0.920	0.685
	seed4_sample2	0.732	0.610	0.923	0.053
	seed4_sample3	0.734	0.643	0.908	0.679
	seed4_sample4	0.708	0.691	0.902	0.685
	seed5_sample0	0.727	0.585	0.921	0.067
	seed5_sample1	0.713	0.664	0.921	0.711
	seed5_sample2	0.679	0.645	0.912	0.710
	seed5_sample3	0.741	0.683	0.910	0.705
	seed5_sample4	0.781	0.621	0.906	0.090
7zko	seed1_sample0	0.508	0.466	0.559	0.405
	seed1_sample1	0.522	0.405	0.717	0.399
	seed1_sample2	0.532	0.470	0.558	0.380
	seed1_sample3	0.546	0.469	0.543	0.568
	seed1_sample4	0.534	0.459	0.537	0.395
	seed2_sample0	0.537	0.455	0.460	0.374
	seed2_sample1	0.532	0.377	0.528	0.416
	seed2_sample2	0.533	0.396	0.533	0.365
	seed2_sample3	0.536	0.406	0.538	0.417
	seed2_sample4	0.550	0.422	0.564	0.405
	seed3_sample0	0.557	0.362	0.553	0.437
	seed3_sample1	0.542	0.476	0.538	0.365
	seed3_sample2	0.522	0.453	0.542	0.529
	seed3_sample3	0.528	0.424	0.535	0.446
	seed3_sample4	0.564	0.335	0.548	0.427
	seed4_sample0	0.551	0.440	0.698	0.562
	seed4_sample1	0.524	0.384	0.544	0.573
	seed4_sample2	0.539	0.501	0.524	0.389
	seed4_sample3	0.564	0.442	0.462	0.411
	seed4_sample4	0.519	0.438	0.430	0.411
	seed5_sample0	0.517	0.365	0.525	0.619
	seed5_sample1	0.552	0.447	0.687	0.375
	seed5_sample2	0.556	0.475	0.535	0.556
	seed5_sample3	0.537	0.424	0.467	0.365
	seed5_sample4	0.536	0.406	0.497	0.357
7zqs	seed1_sample0	0.494	0.313	0.464	0.008
	seed1_sample1	0.490	0.379	0.387	NA
	seed1_sample2	0.349	0.369	0.366	0.000
	seed1_sample3	0.483	0.322	0.349	0.000
	seed1_sample4	0.488	0.331	0.293	0.000
	seed2_sample0	0.596	0.431	0.432	0.018
	seed2_sample1	0.567	0.368	0.360	0.000
	seed2_sample2	0.576	0.383	0.439	NA
	seed2_sample3	0.567	0.315	0.340	0.039
	seed2_sample4	0.560	0.427	0.360	NA
	seed3_sample0	0.427	0.316	0.378	0.021
	seed3_sample1	0.427	0.317	0.381	0.002
	seed3_sample2	0.540	0.376	0.366	0.004
	seed3_sample3	0.516	0.337	0.362	0.019
	seed3_sample4	0.585	0.350	0.385	0.048
	seed4_sample0	0.593	0.389	0.402	NA
	seed4_sample1	0.376	0.376	0.431	0.000
	seed4_sample2	0.395	0.310	0.480	0.029
	seed4_sample3	0.620	0.307	0.322	0.021
	seed4_sample4	0.545	0.380	0.350	0.013
	seed5_sample0	0.371	0.309	0.357	0.009
	seed5_sample1	0.471	0.366	0.375	NA
	seed5_sample2	0.532	0.360	0.348	0.004
	seed5_sample3	0.543	0.330	0.356	0.031
	seed5_sample4	0.337	0.496	0.368	0.020
8d29	seed1_sample0	0.868	0.827	0.923	0.759
	seed1_sample1	0.880	0.842	0.913	0.760
	seed1_sample2	0.884	0.846	0.920	0.772
	seed1_sample3	0.879	0.833	0.925	0.804
	seed1_sample4	0.878	0.826	0.907	0.788
	seed2_sample0	0.880	0.771	0.916	0.762
	seed2_sample1	0.880	0.825	0.913	0.808
	seed2_sample2	0.890	0.820	0.920	0.776

	seed2_sample3	0.874	0.789	0.907	0.800
	seed2_sample4	0.877	0.851	0.923	0.794
	seed3_sample0	0.886	0.857	0.924	0.776
	seed3_sample1	0.882	0.862	0.921	0.756
	seed3_sample2	0.880	0.851	0.918	0.789
	seed3_sample3	0.879	0.784	0.919	0.771
	seed3_sample4	0.876	0.814	0.925	0.776
	seed4_sample0	0.885	0.840	0.920	0.803
	seed4_sample1	0.877	0.820	0.930	0.752
	seed4_sample2	0.875	0.734	0.910	0.776
	seed4_sample3	0.875	0.834	0.906	0.770
	seed4_sample4	0.882	0.821	0.928	0.763
	seed5_sample0	0.886	0.813	0.921	0.791
	seed5_sample1	0.881	0.812	0.924	0.807
	seed5_sample2	0.880	0.767	0.922	0.766
	seed5_sample3	0.874	0.849	0.923	0.780
	seed5_sample4	0.882	0.796	0.921	0.771
8bw5	seed1_sample0	0.552	0.423	0.657	0.544
	seed1_sample1	0.499	0.492	0.643	0.491
	seed1_sample2	0.510	0.481	0.699	0.476
	seed1_sample3	0.581	0.526	0.510	0.403
	seed1_sample4	0.575	0.504	0.685	0.442
	seed2_sample0	0.568	0.514	0.693	0.489
	seed2_sample1	0.576	0.456	0.540	0.485
	seed2_sample2	0.572	0.587	0.482	0.430
	seed2_sample3	0.540	0.482	0.682	0.522
	seed2_sample4	0.462	0.406	0.441	0.466
	seed3_sample0	0.550	0.487	0.682	0.510
	seed3_sample1	0.502	0.453	0.541	0.480
	seed3_sample2	0.491	0.476	0.681	0.475
	seed3_sample3	0.556	0.454	0.538	0.442
	seed3_sample4	0.541	0.484	0.535	0.414
	seed4_sample0	0.590	0.447	0.519	0.495
	seed4_sample1	0.528	0.498	0.509	0.449
	seed4_sample2	0.653	0.476	0.668	0.477
	seed4_sample3	0.617	0.448	0.638	0.485
	seed4_sample4	0.499	0.438	0.611	0.461
	seed5_sample0	0.503	0.493	0.673	0.455
	seed5_sample1	0.621	0.391	0.703	0.505
	seed5_sample2	0.526	0.422	0.658	0.534
	seed5_sample3	0.522	0.512	0.641	0.462
	seed5_sample4	0.489	0.439	0.541	0.478
8tfd	seed1_sample0	0.108	0.161	0.109	0.270
	seed1_sample1	0.155	0.151	0.109	0.274
	seed1_sample2	0.127	0.168	0.201	0.272
	seed1_sample3	0.131	0.162	0.160	0.274
	seed1_sample4	0.133	0.159	0.153	0.271
	seed2_sample0	0.146	0.160	0.108	0.275
	seed2_sample1	0.150	0.170	0.163	0.273
	seed2_sample2	0.145	0.147	0.168	0.271
	seed2_sample3	0.171	0.171	0.132	0.272
	seed2_sample4	0.184	0.160	0.144	0.272
	seed3_sample0	0.151	0.177	0.115	0.273
	seed3_sample1	0.138	0.154	0.201	0.271
	seed3_sample2	0.108	0.155	0.116	0.273
	seed3_sample3	0.191	0.161	0.102	0.271
	seed3_sample4	0.111	0.168	0.203	0.273
	seed4_sample0	0.155	0.167	0.233	0.273
	seed4_sample1	0.208	0.156	0.130	0.267
	seed4_sample2	0.179	0.146	0.160	0.271
	seed4_sample3	0.132	0.186	0.132	0.272
	seed4_sample4	0.143	0.168	0.195	0.269
	seed5_sample0	0.130	0.155	0.152	0.273
	seed5_sample1	0.146	0.164	0.218	0.272
	seed5_sample2	0.138	0.141	0.205	0.276
	seed5_sample3	0.162	0.159	0.152	0.271
	seed5_sample4	0.218	0.175	0.223	0.272
8tqs	seed1_sample0	0.874	0.775	0.899	0.041
	seed1_sample1	0.894	0.758	0.873	0.093

	seed1_sample2	0.907	0.743	0.875	0.086
	seed1_sample3	0.872	0.759	0.883	0.031
	seed1_sample4	0.902	0.685	0.873	0.047
	seed2_sample0	0.896	0.611	0.876	0.072
	seed2_sample1	0.887	0.762	0.865	0.060
	seed2_sample2	0.896	0.724	0.882	0.020
	seed2_sample3	0.886	0.789	0.885	0.091
	seed2_sample4	0.900	0.732	0.871	0.079
	seed3_sample0	0.902	0.764	0.871	0.354
	seed3_sample1	0.893	0.767	0.867	0.061
	seed3_sample2	0.923	0.787	0.869	0.051
	seed3_sample3	0.895	0.784	0.881	0.006
	seed3_sample4	0.918	0.760	0.875	0.042
	seed4_sample0	0.890	0.807	0.870	0.047
	seed4_sample1	0.907	0.604	0.882	0.069
	seed4_sample2	0.881	0.763	0.877	0.114
	seed4_sample3	0.878	0.766	0.873	0.081
	seed4_sample4	0.897	0.757	0.872	0.071
	seed5_sample0	0.887	0.685	0.860	0.072
	seed5_sample1	0.905	0.763	0.873	0.074
	seed5_sample2	0.904	0.742	0.882	0.070
	seed5_sample3	0.877	0.772	0.864	0.025
	seed5_sample4	0.914	0.663	0.869	0.051
8zbf	seed1_sample0	0.368	0.106	0.323	0.165
	seed1_sample1	0.409	0.110	0.276	0.244
	seed1_sample2	0.401	0.114	0.329	0.218
	seed1_sample3	0.397	0.095	0.294	0.133
	seed1_sample4	0.373	0.083	0.388	0.261
	seed2_sample0	0.346	0.117	0.286	0.270
	seed2_sample1	0.392	0.126	0.341	0.255
	seed2_sample2	0.372	0.108	0.378	0.272
	seed2_sample3	0.339	0.091	0.246	0.151
	seed2_sample4	0.309	0.102	0.302	0.162
	seed3_sample0	0.328	0.122	0.270	0.154
	seed3_sample1	0.378	0.082	0.319	0.256
	seed3_sample2	0.396	0.165	0.285	0.145
	seed3_sample3	0.387	0.095	0.278	0.196
	seed3_sample4	0.315	0.138	0.366	0.272
	seed4_sample0	0.326	0.137	0.322	0.204
	seed4_sample1	0.368	0.104	0.299	0.158
	seed4_sample2	0.321	0.083	0.326	0.251
	seed4_sample3	0.343	0.095	0.294	0.244
	seed4_sample4	0.326	0.160	0.276	0.257
	seed5_sample0	0.356	0.097	0.264	0.263
	seed5_sample1	0.351	0.113	0.339	0.254
	seed5_sample2	0.345	0.085	0.226	0.253
	seed5_sample3	0.392	0.088	0.333	0.252
	seed5_sample4	0.380	0.147	0.289	0.140
9gxh	seed1_sample0	0.234	0.124	0.218	0.069
	seed1_sample1	0.184	0.106	0.222	0.068
	seed1_sample2	0.189	0.110	0.219	0.063
	seed1_sample3	0.158	0.151	0.224	0.070
	seed1_sample4	0.203	0.094	0.214	0.066
	seed2_sample0	0.176	0.112	0.256	0.066
	seed2_sample1	0.204	0.120	0.221	0.073
	seed2_sample2	0.152	0.114	0.208	0.074
	seed2_sample3	0.195	0.110	0.160	0.063
	seed2_sample4	0.210	0.118	0.234	0.070
	seed3_sample0	0.113	0.110	0.188	0.058
	seed3_sample1	0.204	0.116	0.159	0.074
	seed3_sample2	0.214	0.126	0.224	0.074
	seed3_sample3	0.178	0.102	0.236	0.060
	seed3_sample4	0.194	0.099	0.221	0.059
	seed4_sample0	0.183	0.129	0.215	0.092
	seed4_sample1	0.148	0.135	0.212	0.074
	seed4_sample2	0.180	0.104	0.235	0.063
	seed4_sample3	0.187	0.111	0.246	0.065
	seed4_sample4	0.190	0.104	0.127	0.061
	seed5_sample0	0.185	0.113	0.234	0.059

seed5_sample1	0.158	0.100	0.217	0.064
seed5_sample2	0.213	0.117	0.215	0.079
seed5_sample3	0.166	0.115	0.226	0.063
seed5_sample4	0.147	0.122	0.194	0.073

Table S4. Interfacial H-bond counts in top-ranked predictions for each protein-aptamer complex. Values are reported for ground-truth (GT) structures and the four models. Complex names in red indicate cases absent from the Boltz-2 training set.

Complex	AF3	Chai-1	Boltz-2	RF2NA	GT
7lri	14	10	11	0	15
7szu	7	6	4	2	8
7v5n	0	5	10	0	12
7zko	9	5	4	1	9
7zqs	10	14	16	12	19
8d29	7	4	9	6	8
8bw5	1	6	9	2	5
8tfd	3	5	2	1	8
8tqs	4	7	2	0	8
8zbf	7	5	8	5	15
9gxh	0	10	2	2	8

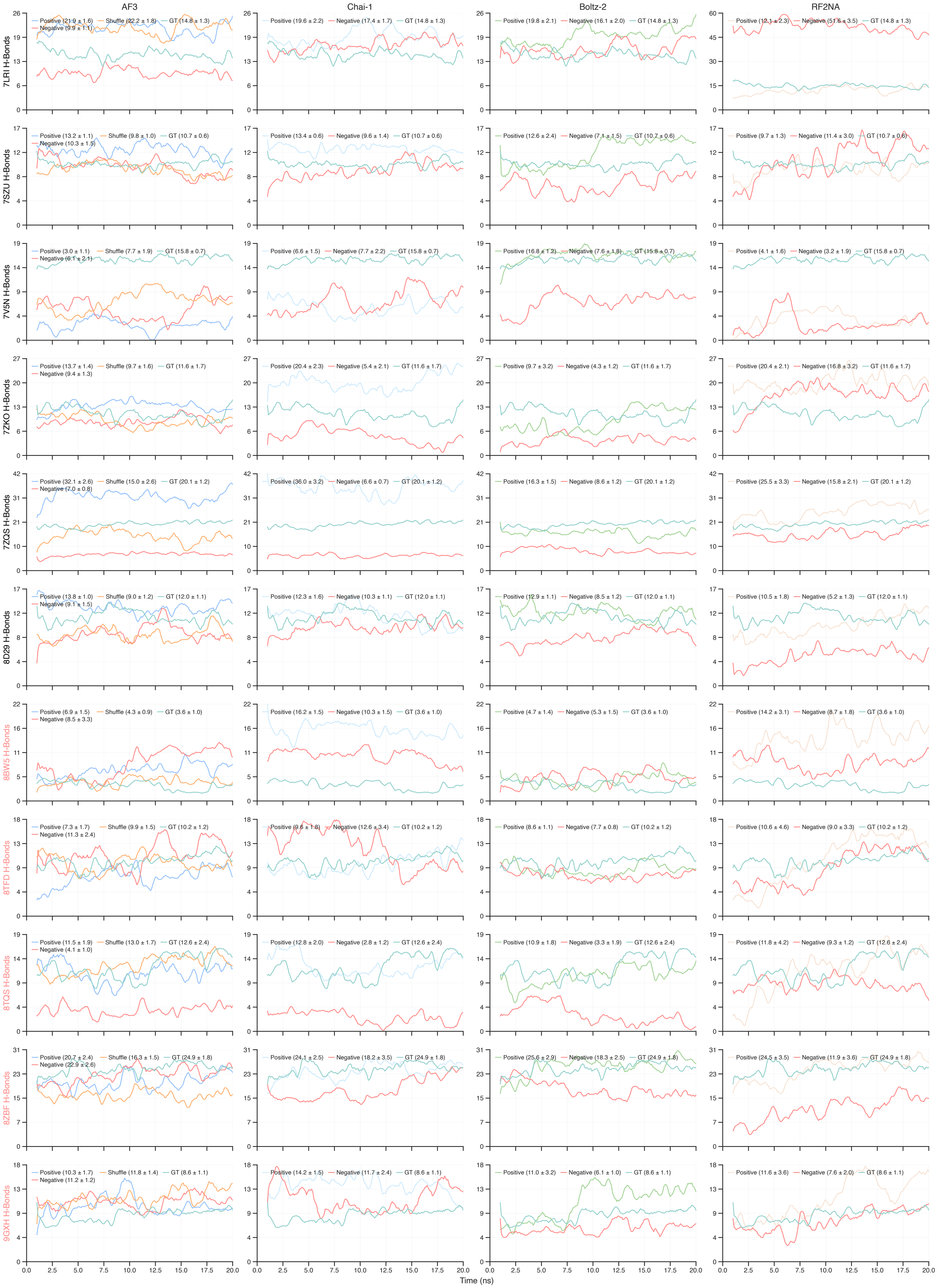

Fig. S3. Interfacial H-bond analysis of benchmark protein-aptamer complexes over 20 ns MD simulations. Results are shown for GT structures and the top-ranked predictions from four models. Positive (native sequence pairs), Negative (intentionally mismatched pairs), and Shuffle (cognate proteins paired with shuffled native aptamers) are shown; Shuffle was calculated only for AF3. Values in parentheses denote mean ± standard deviation. Complex names in red indicate cases absent from the Boltz-2 training set.

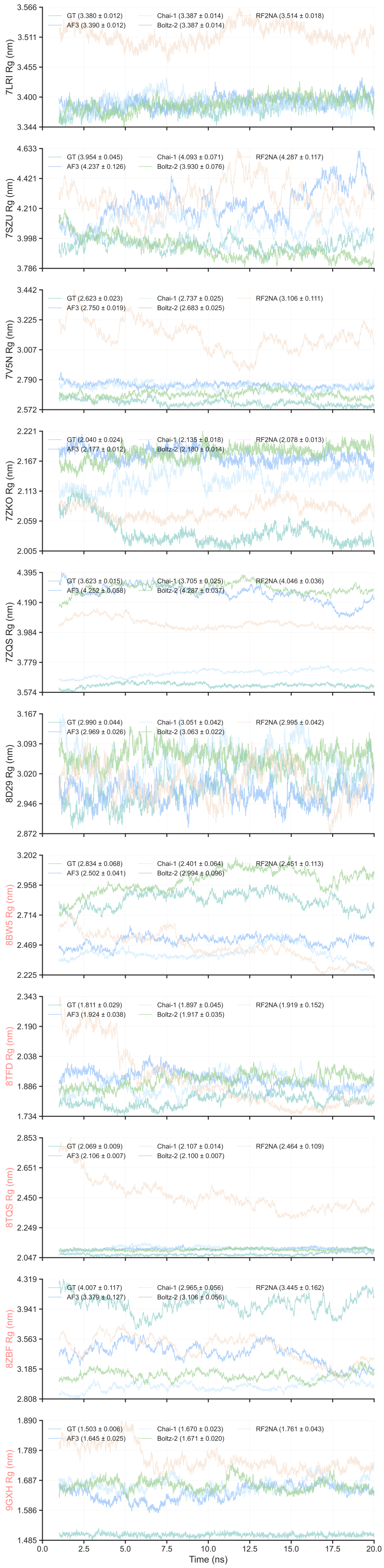

Fig. S4. Radius of gyration (Rg) profiles of benchmark protein-aptamer complexes over 20 ns MD simulations. Results are shown for GT structures and the top-ranked predictions from the four models. Rg was calculated for the combined protein-aptamer complex. Values in parentheses denote mean ± standard deviation. Complex names in red indicate cases absent from the Boltz-2 training set.

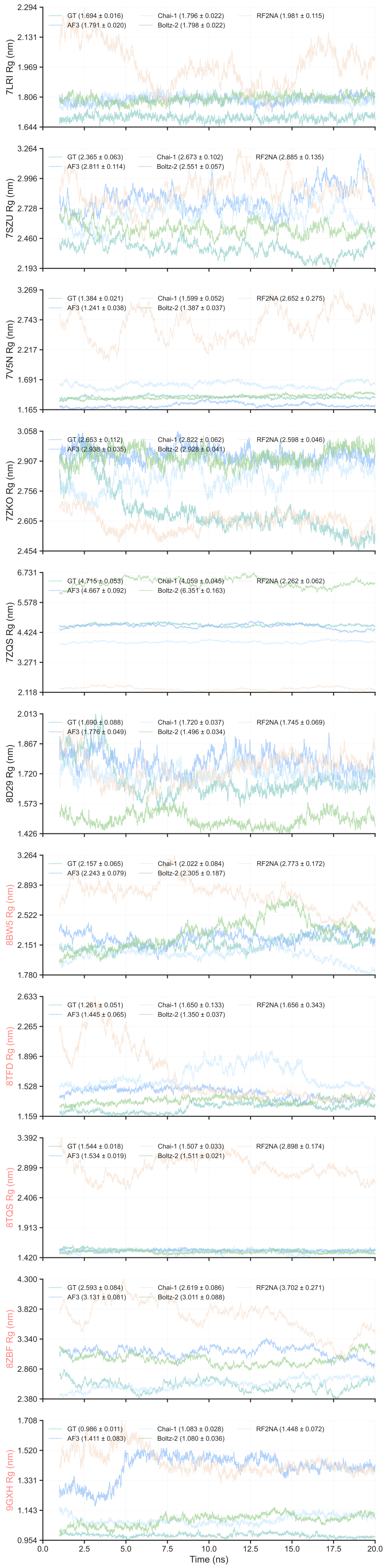

Fig. S5. Rg profiles of aptamers within benchmark protein-aptamer complexes over 20 ns MD simulations. Results are shown for GT structures and the top-ranked predictions from the four models. Rg was calculated for the aptamer component within the complex. Values in parentheses denote mean ± standard deviation. Complex names in red indicate cases absent from the Boltz-2 training set.

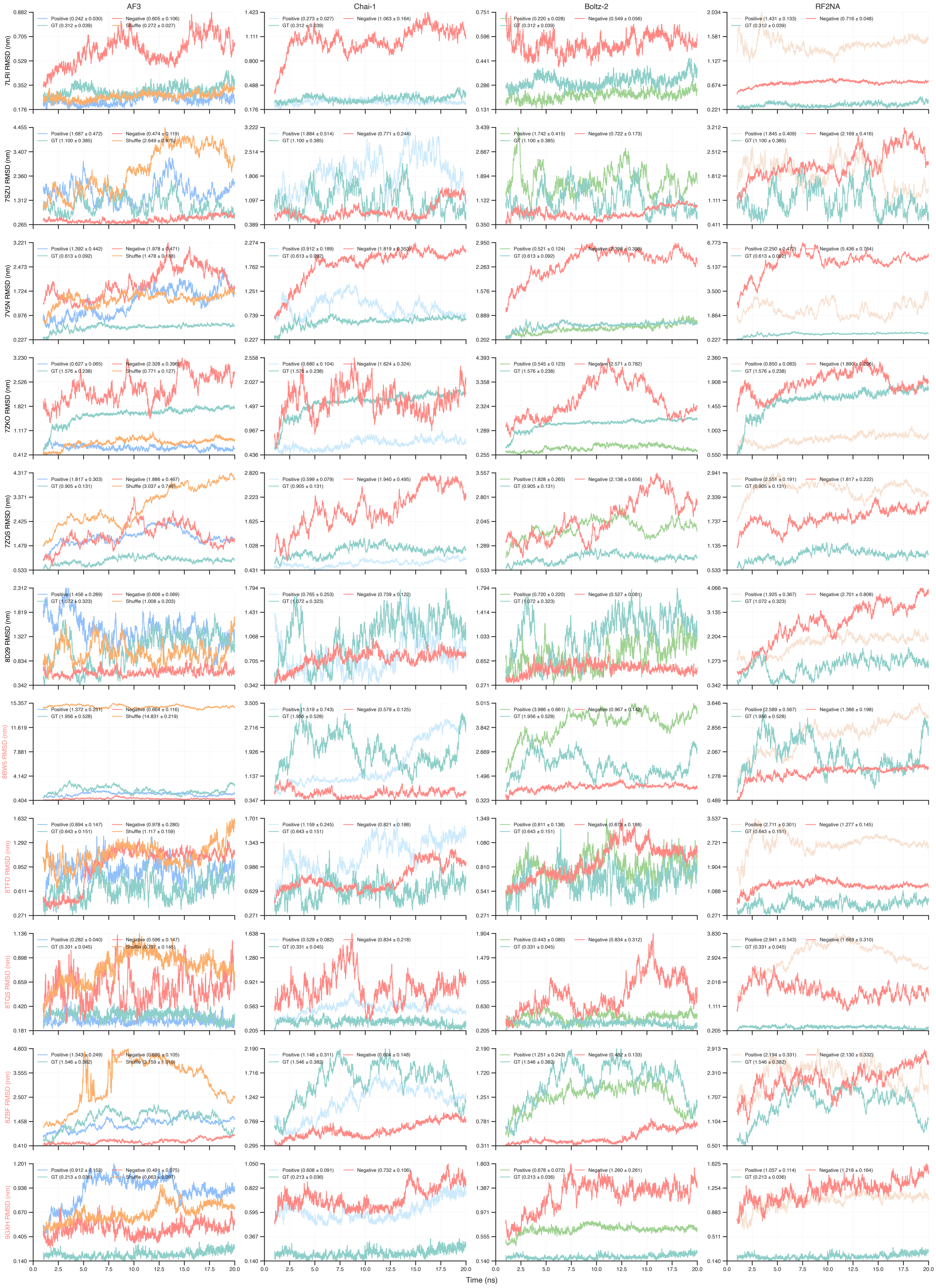

Fig. S6. Root mean square deviation (RMSD) profiles of aptamers after protein-backbone alignment. Results are shown for GT structures and the top-ranked predictions from the four models. Positive (native sequence pairs), Negative (intentionally mismatched pairs), and Shuffle (cognate proteins with shuffled native aptamers) are shown; Shuffle was calculated only for AF3. Values in parentheses denote mean ± standard deviation. Complex names in red indicate cases absent from the Boltz-2 training set.

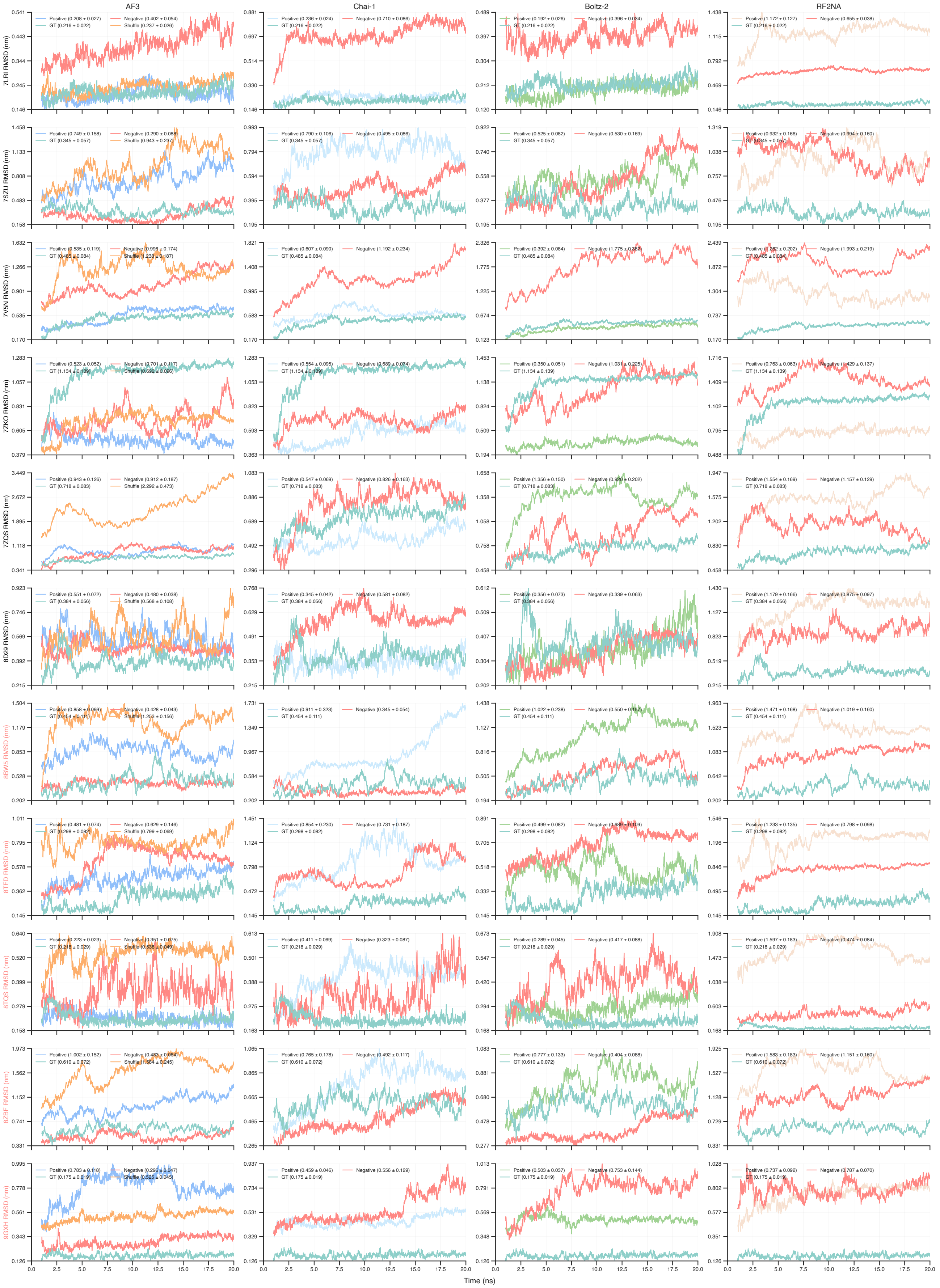

Fig. S7. RMSD profiles of aptamers within benchmark protein-aptamer complexes. Results are shown for GT structures and the top-ranked predictions from the four models. RMSD was calculated for the aptamer component. Positive (native sequence pairs), Negative (intentionally mismatched pairs), and Shuffle (cognate proteins with shuffled native aptamers) are shown; Shuffle was calculated only for AF3. Values in parentheses denote mean ± standard deviation. Complex names in red indicate cases absent from the Boltz-2 training set.

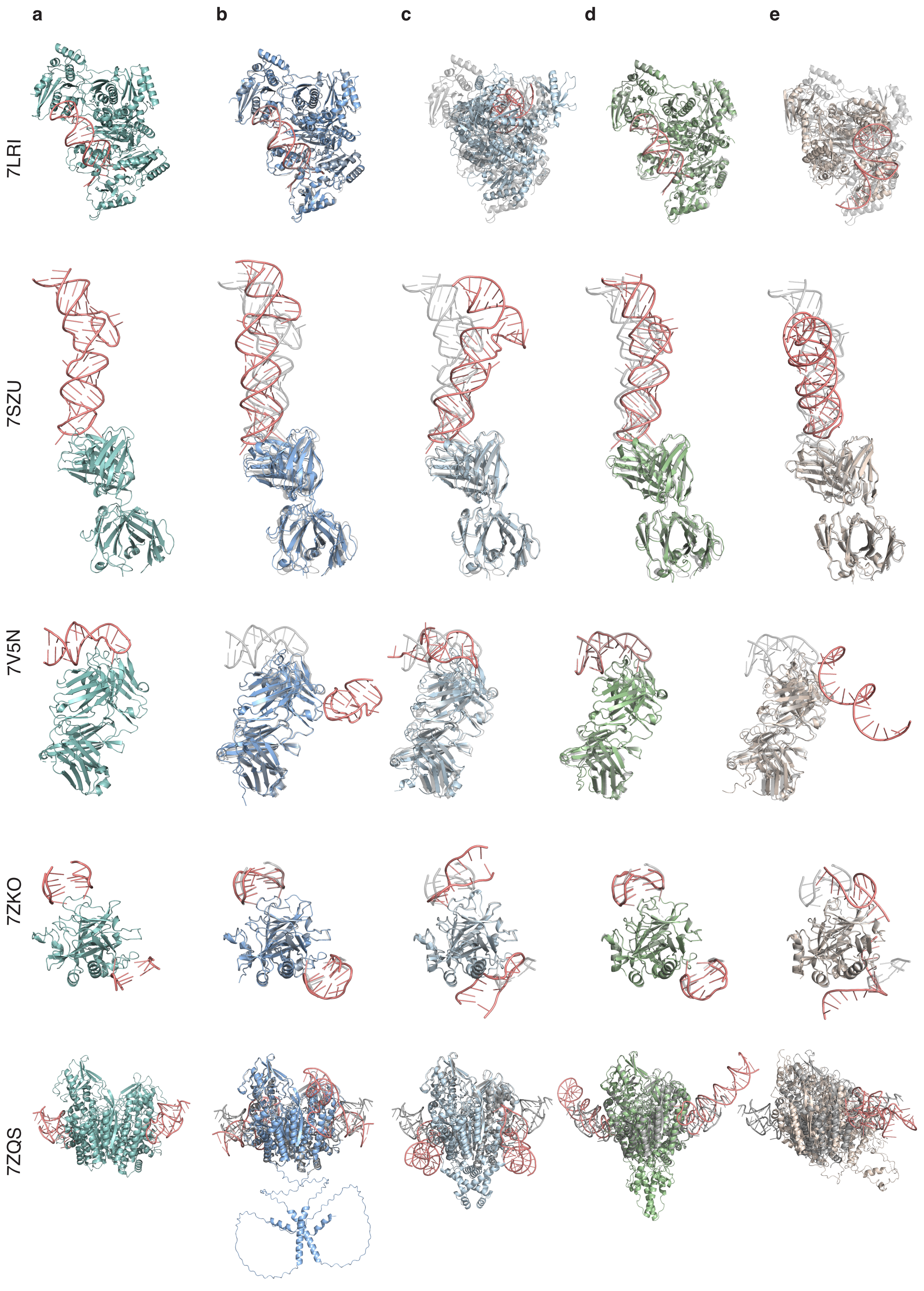

Fig. S8. Visualization of predicted protein-aptamer complexes. Structures are shown for (a) the GT references, and the top-ranked predictions from (b) AF3, (c) Chai-1, (d) Boltz-2, and (e) RF2NA. All predictions were aligned to the GT, which is shown in grey in the overlays. The remaining complexes are shown in Fig. S9.

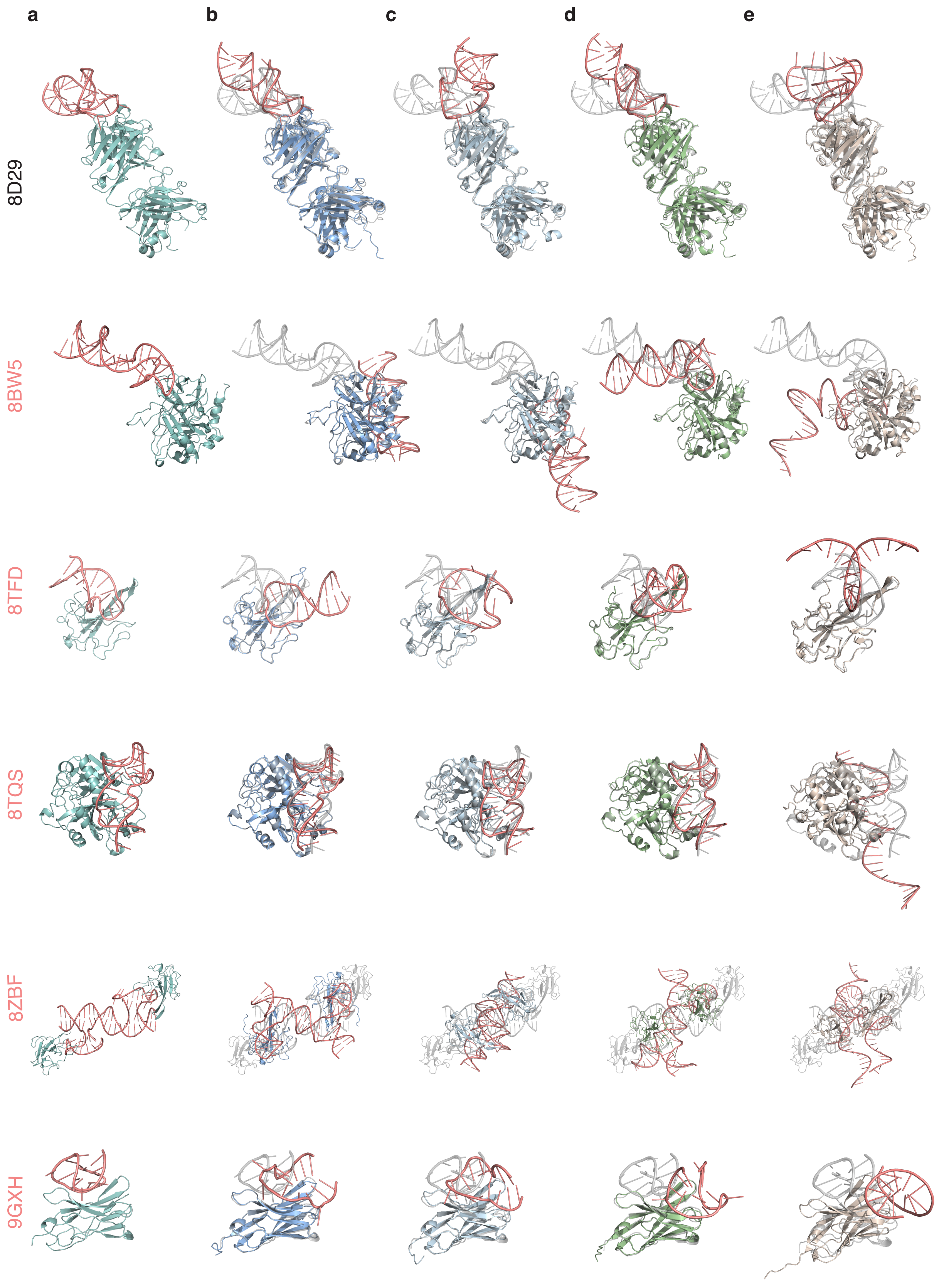

Fig. S9. Visualization of predicted protein-aptamer complexes. Structures are shown for (a) the GT references and the top-ranked predictions from (b) AF3, (c) Chai-1, (d) Boltz-2, and (e) RF2NA. All predictions were aligned to the GT, which is shown in grey in the overlays. Complex names in red indicate cases absent from the Boltz-2 training set.

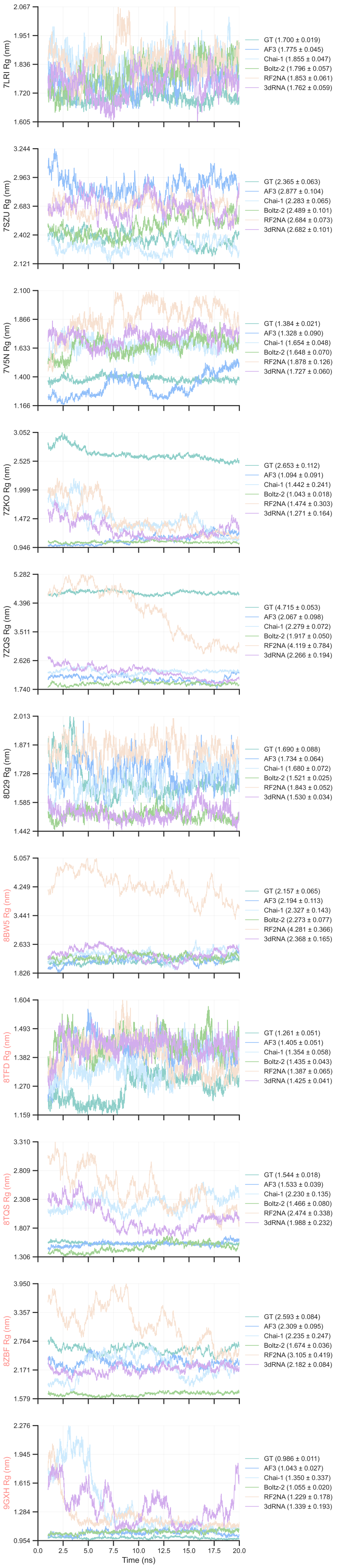

Fig. S10. Rg of aptamer-only structures over 20 ns MD simulations. Aptamer sequences were extracted from experimental protein-aptamer complexes and used to generate aptamer-only predictions. GT corresponds to the aptamer component within the experimental complex, with Rg values taken directly from the in-complex aptamer, serving as a bound-state reference rather than a representation of isolated aptamer behavior. Values in parentheses denote mean ± standard deviation. Complex names in red indicate cases absent from the Boltz-2 training set.

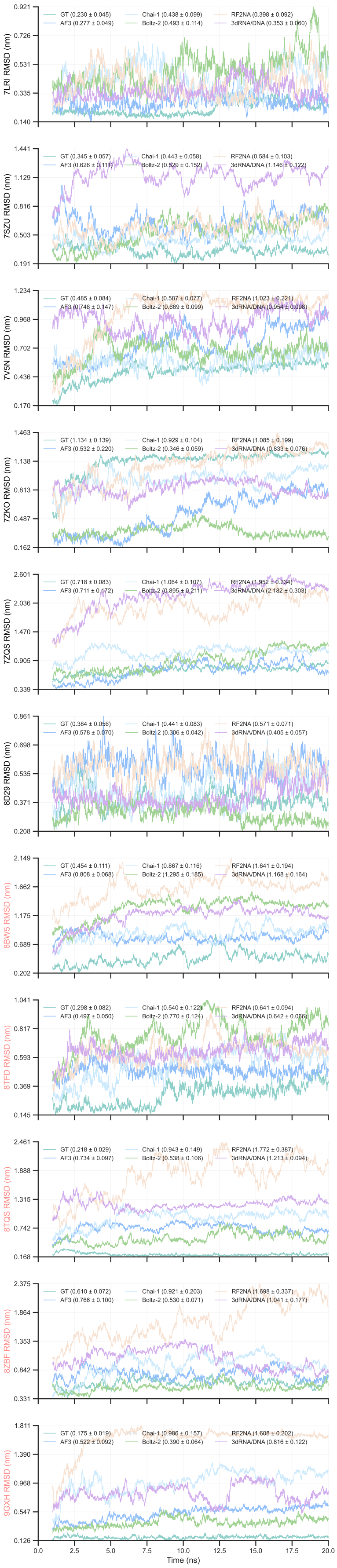

Fig. S11. RMSD of aptamer-only structures over 20 ns MD simulations. Aptamer sequences were extracted from experimental protein-aptamer complexes and used to generate aptamer-only predictions. GT corresponds to the aptamer component within the experimental complex, with RMSD values taken directly from the in-complex aptamer, serving as a bound-state reference rather than a representation of isolated aptamer behavior. Values in parentheses denote mean ± standard deviation. Complex names in red indicate cases absent from the Boltz-2 training set.

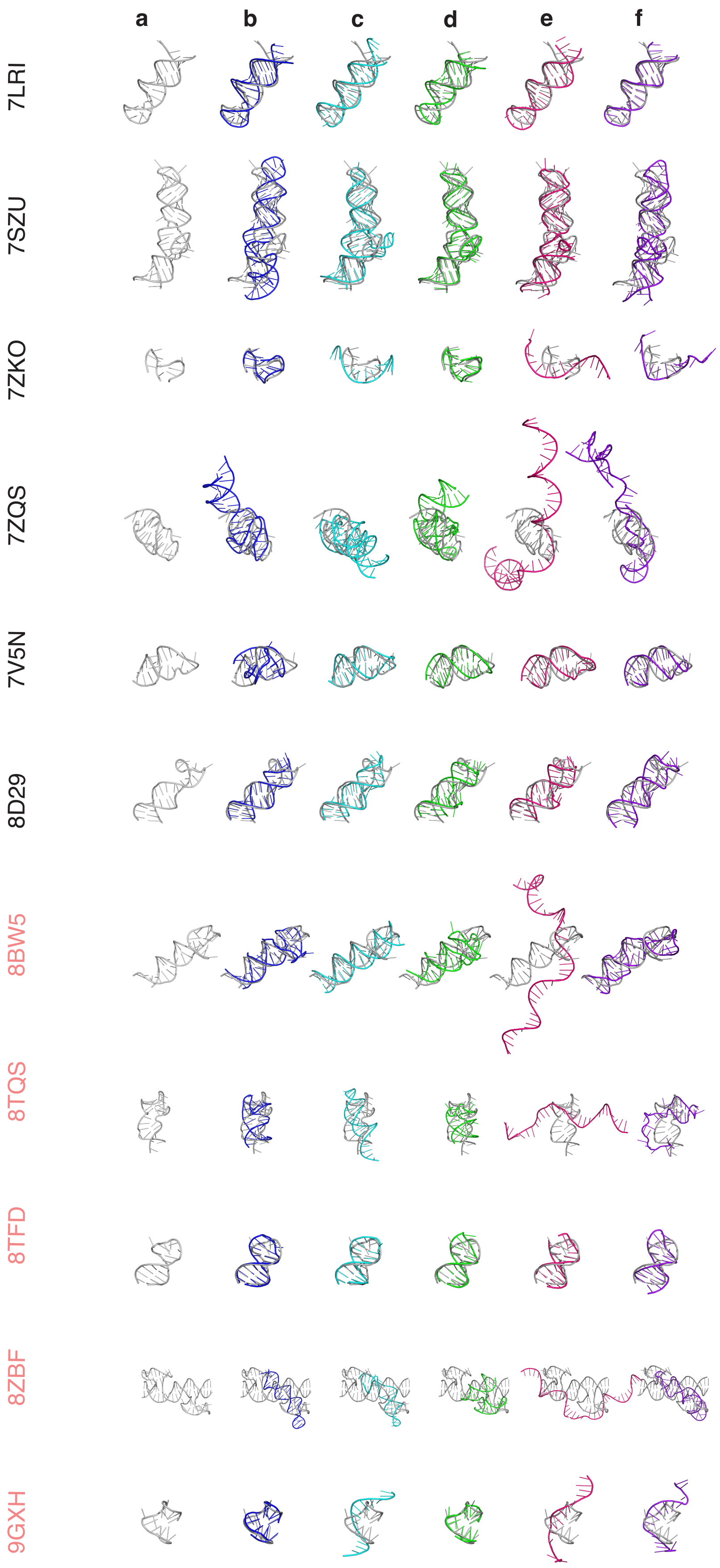

Fig. S12. Visualization of predicted aptamers in isolation. For each case, the label corresponds to the parent protein-aptamer complex, although only the aptamer component is shown. Structures are shown for (a) the GT references and the top-predictions from (b) AF3, (c) Chai-1, (d) Boltz-2, (e) RF2NA, and (f) 3dRNA/DNA. All predictions were aligned to the GT reference, which is shown in grey in the overlays. Complex names in red indicate cases absent from the Boltz-2 training set.

Table S5. Paired *t*-test for iLDDT with and without ions.

Model	Without ion	With ion	Δ Mean	p	Sig.
AF3	0.626 ± 0.251	0.622 ± 0.248	$+0.004$	0.152	ns
Chai-1	0.524 ± 0.284	0.523 ± 0.238	$+0.001$	0.845	ns
Boltz-2	0.623 ± 0.267	0.632 ± 0.259	-0.009	0.029	*

Note: Mean ± standard deviation are reported for iLDDT across 25 predictions per complex. Δ Mean represents the difference between ion-free and ion-included conditions. Results are shown for AF3, Chai-1, and Boltz-2. Significance: *$p < 0.05$; **$p < 0.01$; ***$p < 0.001$; ns, not significant.

Table S6. iLDDT values for 25 ion-included predictions per complex. AF3, Chai-1, and Boltz-2 were each run with five seeds and five samples per seed, yielding 25 predictions per complex. Complex names in red indicate cases absent from the Boltz-2 training set.

Complex	ID	AF3	Chai-1	Boltz-2
7lri	seed1_sample0	0.837	0.754	0.845
	seed1_sample1	0.842	0.771	0.839
	seed1_sample2	0.846	0.790	0.840
	seed1_sample3	0.843	0.788	0.841
	seed1_sample4	0.841	0.718	0.841
	seed2_sample0	0.844	0.776	0.843
	seed2_sample1	0.844	0.691	0.843
	seed2_sample2	0.841	0.693	0.839
	seed2_sample3	0.838	0.675	0.841
	seed2_sample4	0.842	0.687	0.841
	seed3_sample0	0.845	0.686	0.845
	seed3_sample1	0.845	0.771	0.841
	seed3_sample2	0.839	0.696	0.837
	seed3_sample3	0.849	0.692	0.843
	seed3_sample4	0.841	0.697	0.840
	seed4_sample0	0.840	0.694	0.839
	seed4_sample1	0.842	0.678	0.844
	seed4_sample2	0.844	0.777	0.841
	seed4_sample3	0.844	0.760	0.843
	seed4_sample4	0.843	0.706	0.841
	seed5_sample0	0.840	0.767	0.840
	seed5_sample1	0.846	0.678	0.841
	seed5_sample2	0.841	0.785	0.844
	seed5_sample3	0.837	0.684	0.839
	seed5_sample4	0.843	0.776	0.839
7szu	seed1_sample0	0.892	0.872	0.934
	seed1_sample1	0.885	0.881	0.927
	seed1_sample2	0.886	0.873	0.922
	seed1_sample3	0.880	0.871	0.928
	seed1_sample4	0.905	0.884	0.931
	seed2_sample0	0.909	0.879	0.941
	seed2_sample1	0.896	0.869	0.944
	seed2_sample2	0.886	0.872	0.949
	seed2_sample3	0.877	0.877	0.936
	seed2_sample4	0.880	0.878	0.927
	seed3_sample0	0.901	0.884	0.937
	seed3_sample1	0.901	0.859	0.941
	seed3_sample2	0.885	0.884	0.924
	seed3_sample3	0.881	0.884	0.927
	seed3_sample4	0.885	0.877	0.927
	seed4_sample0	0.893	0.881	0.944
	seed4_sample1	0.917	0.874	0.927
	seed4_sample2	0.880	0.883	0.927
	seed4_sample3	0.899	0.864	0.924
	seed4_sample4	0.885	0.878	0.928
	seed5_sample0	0.877	0.868	0.936
	seed5_sample1	0.887	0.872	0.938
	seed5_sample2	0.902	0.871	0.938
	seed5_sample3	0.884	0.866	0.932
	seed5_sample4	0.884	0.866	0.926
7zko	seed1_sample0	0.520	0.455	0.528
	seed1_sample1	0.524	0.437	0.540
	seed1_sample2	0.533	0.463	0.543
	seed1_sample3	0.521	0.442	0.468
	seed1_sample4	0.528	0.475	0.391
	seed2_sample0	0.528	0.477	0.544
	seed2_sample1	0.529	0.464	0.545
	seed2_sample2	0.528	0.465	0.542
	seed2_sample3	0.524	0.589	0.462
	seed2_sample4	0.531	0.442	0.506
	seed3_sample0	0.527	0.454	0.536
	seed3_sample1	0.465	0.464	0.540
	seed3_sample2	0.517	0.478	0.460
	seed3_sample3	0.529	0.460	0.444

	seed3_sample4	0.539	0.449	0.456
	seed4_sample0	0.534	0.453	0.690
	seed4_sample1	0.521	0.468	0.560
	seed4_sample2	0.531	0.457	0.696
	seed4_sample3	0.521	0.455	0.457
	seed4_sample4	0.516	0.452	0.507
	seed5_sample0	0.513	0.484	0.682
	seed5_sample1	0.545	0.431	0.527
	seed5_sample2	0.534	0.440	0.498
	seed5_sample3	0.529	0.482	0.491
	seed5_sample4	0.526	0.442	0.452
7zqs	seed1_sample0	0.540	0.364	0.399
	seed1_sample1	0.529	0.364	0.388
	seed1_sample2	0.550	0.353	0.380
	seed1_sample3	0.592	0.355	0.507
	seed1_sample4	0.564	0.322	0.300
	seed2_sample0	0.530	0.390	0.409
	seed2_sample1	0.503	0.343	0.472
	seed2_sample2	0.546	0.364	0.340
	seed2_sample3	0.562	0.364	0.435
	seed2_sample4	0.574	0.424	0.407
	seed3_sample0	0.444	0.428	0.443
	seed3_sample1	0.382	0.358	0.404
	seed3_sample2	0.554	0.363	0.369
	seed3_sample3	0.721	0.342	0.401
	seed3_sample4	0.594	0.380	0.336
	seed4_sample0	0.592	0.325	0.395
	seed4_sample1	0.395	0.320	0.463
	seed4_sample2	0.377	0.386	0.434
	seed4_sample3	0.591	0.299	0.350
	seed4_sample4	0.387	0.349	0.337
	seed5_sample0	0.344	0.333	0.377
	seed5_sample1	0.479	0.446	0.410
	seed5_sample2	0.573	0.409	0.368
	seed5_sample3	0.545	0.404	0.477
	seed5_sample4	0.312	0.475	0.425
8d29	seed1_sample0	0.869	0.682	0.895
	seed1_sample1	0.883	0.691	0.898
	seed1_sample2	0.883	0.641	0.885
	seed1_sample3	0.880	0.751	0.891
	seed1_sample4	0.880	0.690	0.897
	seed2_sample0	0.884	0.697	0.912
	seed2_sample1	0.879	0.709	0.897
	seed2_sample2	0.890	0.700	0.887
	seed2_sample3	0.874	0.717	0.889
	seed2_sample4	0.878	0.695	0.878
	seed3_sample0	0.886	0.704	0.898
	seed3_sample1	0.883	0.706	0.890
	seed3_sample2	0.878	0.685	0.896
	seed3_sample3	0.880	0.674	0.884
	seed3_sample4	0.877	0.695	0.903
	seed4_sample0	0.884	0.719	0.907
	seed4_sample1	0.879	0.750	0.905
	seed4_sample2	0.881	0.757	0.905
	seed4_sample3	0.876	0.711	0.886
	seed4_sample4	0.880	0.706	0.877
	seed5_sample0	0.884	0.712	0.904
	seed5_sample1	0.881	0.692	0.906
	seed5_sample2	0.879	0.667	0.899
	seed5_sample3	0.874	0.694	0.893
	seed5_sample4	0.882	0.677	0.907
8bw5	seed1_sample0	0.463	0.619	0.753
	seed1_sample1	0.464	0.594	0.721
	seed1_sample2	0.468	0.545	0.676
	seed1_sample3	0.451	0.663	0.661
	seed1_sample4	0.471	0.590	0.727
	seed2_sample0	0.516	0.546	0.686
	seed2_sample1	0.446	0.512	0.726
	seed2_sample2	0.506	0.598	0.712

	seed2_sample3	0.452	0.612	0.727
	seed2_sample4	0.542	0.531	0.684
	seed3_sample0	0.451	0.553	0.713
	seed3_sample1	0.526	0.673	0.671
	seed3_sample2	0.503	0.584	0.651
	seed3_sample3	0.420	0.679	0.685
	seed3_sample4	0.447	0.539	0.550
	seed4_sample0	0.409	0.504	0.706
	seed4_sample1	0.452	0.574	0.748
	seed4_sample2	0.502	0.570	0.678
	seed4_sample3	0.539	0.562	0.615
	seed4_sample4	0.463	0.587	0.534
	seed5_sample0	0.506	0.645	0.738
	seed5_sample1	0.522	0.569	0.650
	seed5_sample2	0.530	0.629	0.739
	seed5_sample3	0.429	0.591	0.464
	seed5_sample4	0.470	0.574	0.590
8tqs	seed1_sample0	0.886	0.637	0.880
	seed1_sample1	0.890	0.641	0.879
	seed1_sample2	0.898	0.786	0.879
	seed1_sample3	0.875	0.649	0.879
	seed1_sample4	0.897	0.635	0.879
	seed2_sample0	0.904	0.642	0.883
	seed2_sample1	0.886	0.617	0.888
	seed2_sample2	0.883	0.644	0.875
	seed2_sample3	0.882	0.644	0.877
	seed2_sample4	0.887	0.635	0.877
	seed3_sample0	0.890	0.628	0.872
	seed3_sample1	0.890	0.631	0.879
	seed3_sample2	0.913	0.635	0.882
	seed3_sample3	0.891	0.646	0.881
	seed3_sample4	0.894	0.646	0.886
	seed4_sample0	0.890	0.621	0.878
	seed4_sample1	0.889	0.668	0.888
	seed4_sample2	0.871	0.631	0.881
	seed4_sample3	0.876	0.637	0.876
	seed4_sample4	0.887	0.673	0.875
	seed5_sample0	0.881	0.604	0.873
	seed5_sample1	0.900	0.618	0.899
	seed5_sample2	0.897	0.602	0.873
	seed5_sample3	0.881	0.648	0.891
	seed5_sample4	0.883	0.646	0.671
8zbf	seed1_sample0	0.322	0.108	0.244
	seed1_sample1	0.319	0.121	0.336
	seed1_sample2	0.418	0.096	0.334
	seed1_sample3	0.315	0.085	0.368
	seed1_sample4	0.389	0.126	0.374
	seed2_sample0	0.419	0.168	0.327
	seed2_sample1	0.413	0.133	0.287
	seed2_sample2	0.404	0.139	0.323
	seed2_sample3	0.412	0.098	0.310
	seed2_sample4	0.407	0.161	0.290
	seed3_sample0	0.368	0.146	0.400
	seed3_sample1	0.450	0.154	0.352
	seed3_sample2	0.330	0.097	0.274
	seed3_sample3	0.405	0.122	0.375
	seed3_sample4	0.314	0.075	0.294
	seed4_sample0	0.381	0.131	0.255
	seed4_sample1	0.422	0.130	0.358
	seed4_sample2	0.446	0.085	0.316
	seed4_sample3	0.420	0.100	0.329
	seed4_sample4	0.395	0.157	0.300
	seed5_sample0	0.425	0.086	0.316
	seed5_sample1	0.362	0.115	0.224
	seed5_sample2	0.366	0.116	0.337
	seed5_sample3	0.391	0.103	0.325
	seed5_sample4	0.338	0.133	0.331
9gxh	seed1_sample0	0.172	0.204	0.214
	seed1_sample1	0.202	0.245	0.249

seed1_sample2	0.209	0.240	0.258
seed1_sample3	0.165	0.236	0.252
seed1_sample4	0.204	0.236	0.264
seed2_sample0	0.193	0.277	0.212
seed2_sample1	0.217	0.210	0.221
seed2_sample2	0.207	0.235	0.257
seed2_sample3	0.220	0.192	0.202
seed2_sample4	0.192	0.143	0.261
seed3_sample0	0.209	0.275	0.222
seed3_sample1	0.236	0.237	0.201
seed3_sample2	0.243	0.212	0.211
seed3_sample3	0.188	0.189	0.253
seed3_sample4	0.132	0.289	0.256
seed4_sample0	0.194	0.193	0.218
seed4_sample1	0.166	0.168	0.227
seed4_sample2	0.195	0.229	0.217
seed4_sample3	0.202	0.156	0.208
seed4_sample4	0.191	0.266	0.263
seed5_sample0	0.240	0.254	0.209
seed5_sample1	0.148	0.263	0.190
seed5_sample2	0.213	0.197	0.256
seed5_sample3	0.167	0.166	0.246
seed5_sample4	0.161	0.245	0.255

Table S7. ΔG_{bind} of the top-ranked predictions for each protein-aptamer complex with ions. Values are reported in kcal mol^{-1} for AF3, Chai-1, Boltz-2, and GT. Complex names in red indicate cases absent from the Boltz-2 training set.

Complex	AF3	Chai-1	Boltz-2	GT
7lri	-79.44	-34.58	-84.53	-44.66
7szu	-78.02	-98.66	-62.32	-64.06
7zko	-54.08	-60.57	-69.97	-68.79
7zqs	-123.91	-59.97	-66.77	-98.05
8d29	-79.49	-61.46	-77.49	-78.73
8bw5	-15.80	-37.36	-39.78	-27.36
8tqs	-91.75	-42.53	-87.60	-80.01
8zbf	-167.88	-111.95	-167.45	-143.79
9gxh	-54.70	-53.25	-35.24	-48.79

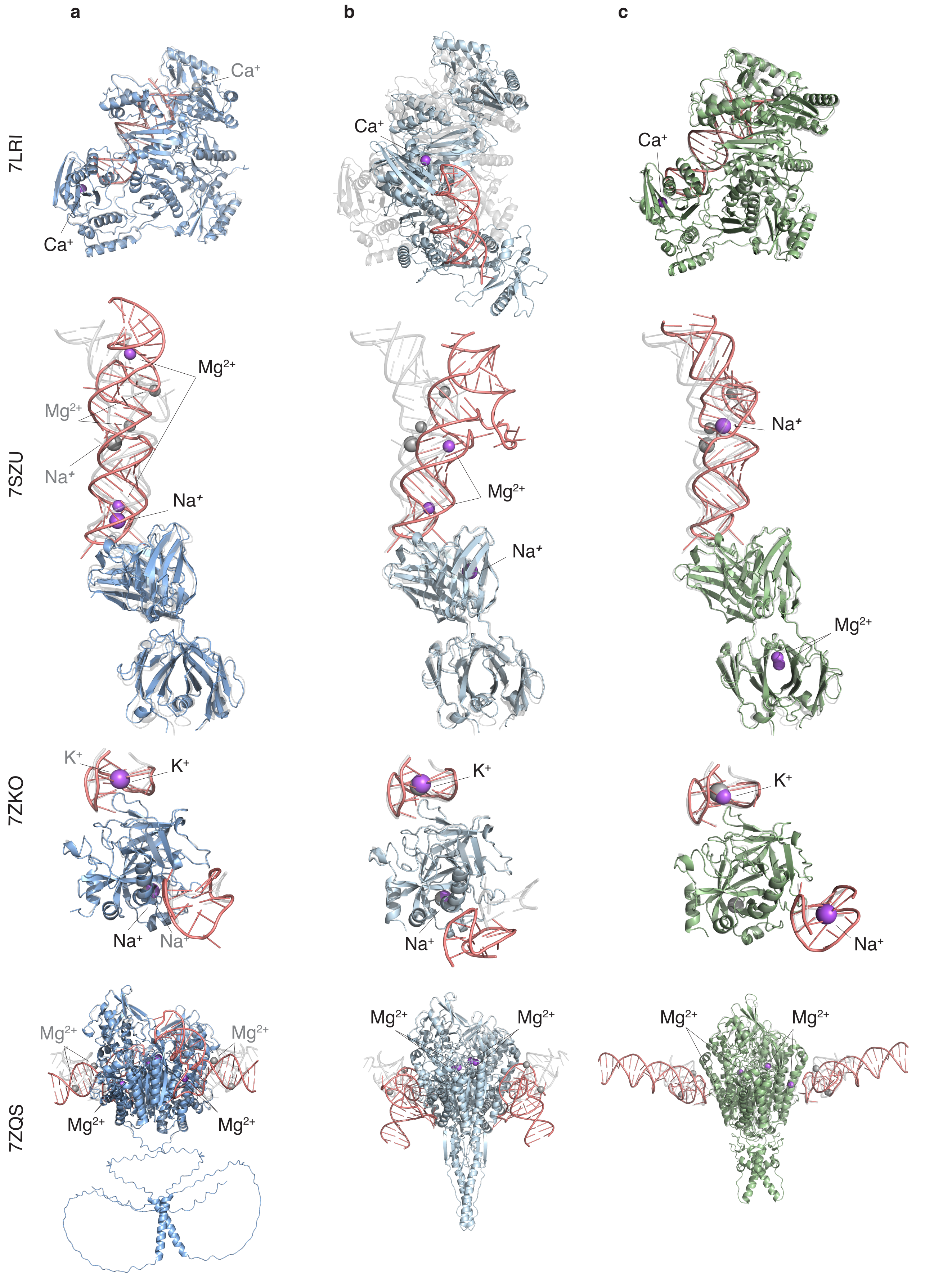

Fig. S13. Predicted protein-aptamer complexes with ions. For each complex, structures are shown for the top-ranked predictions from (a) AF3, (b) Chai-1, and (c) Boltz-2. All predicted structures were aligned to the GT structure, which is shown in grey in the overlays. Predicted ions are shown in purple, and GT ions are shown and labeled in grey.

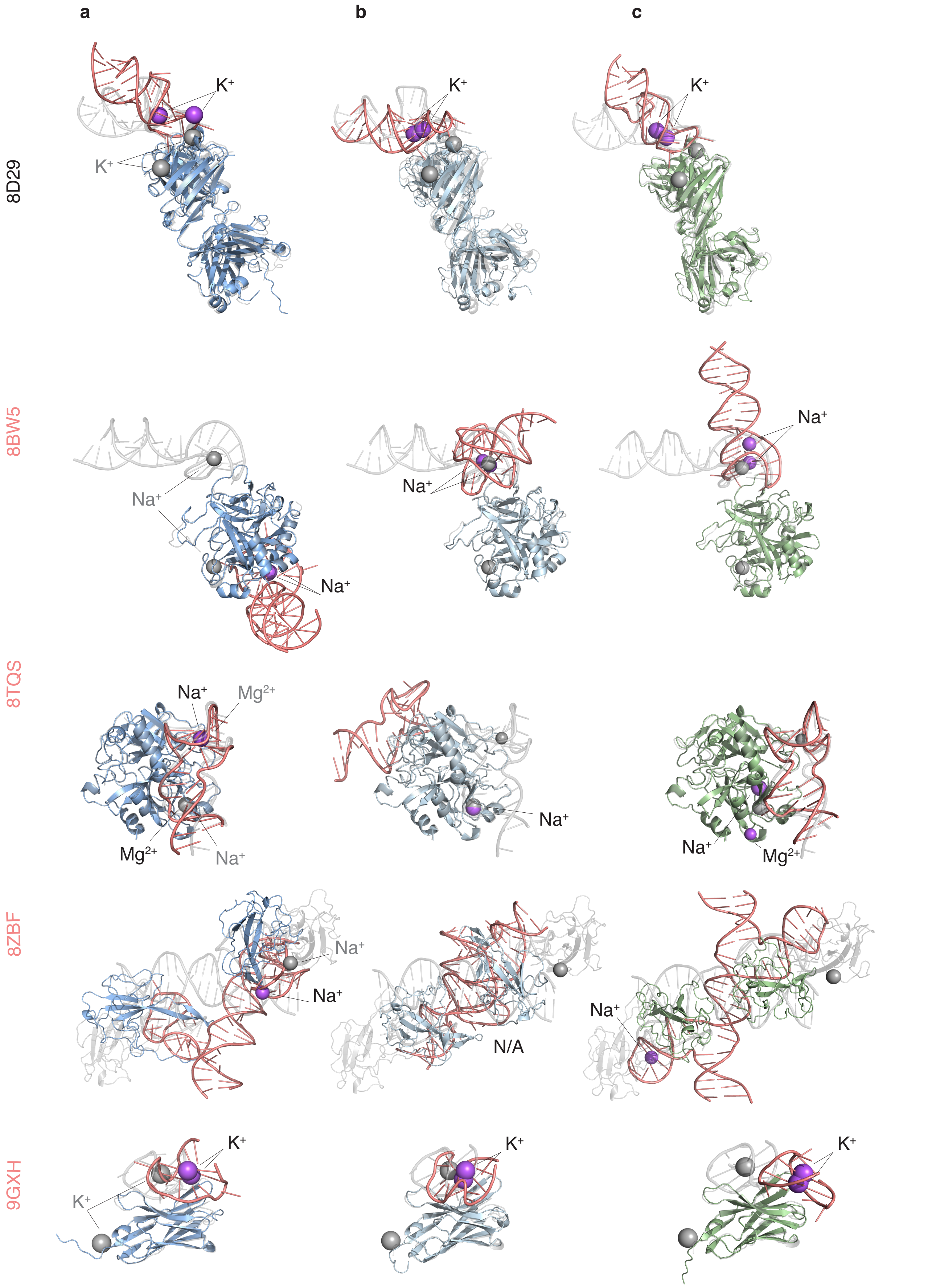

Fig. S14. Predicted protein-aptamer complexes with ions. For each complex, structures are shown for the top-ranked predictions from (a) AF3, (b) Chai-1, and (c) Boltz-2. All predicted structures were aligned to the GT structure, which is shown in grey in the overlays. Predicted ions are shown in purple, and GT ions are shown and labeled in grey. Complex names in red indicate cases absent from the Boltz-2 training set.

Table S8. PyMOL-based RMSD values for aptamers. Values were obtained by aligning the GT aptamer structures, extracted from the experimental protein-aptamer complexes, to the top-ranked predictions from five models. All alignments were performed in PyMOL with refinement cycles disabled. Complex names in red indicate cases absent from the Boltz-2 training set.

Complex	AF3	Chai-1	Boltz-2	RF2NA	3dRNA/DNA
7lri	4.304	7.059	5.100	4.785	2.629
7szu	9.310	7.243	2.560	5.401	8.013
7v5n	11.756	3.579	3.871	4.009	4.256
7zko	3.187	12.654	3.245	16.912	12.552
7zqs	10.016	13.969	11.297	28.993	27.042
8d29	6.810	7.282	6.632	5.137	6.530
8bw5	8.877	8.113	10.855	38.168	8.982
8tfd	3.920	4.783	3.966	3.773	4.325
8tqs	7.617	15.600	9.950	30.455	18.533
8zbf	22.101	22.057	18.659	23.319	19.070
9gxh	2.832	12.019	2.312	15.673	11.844

Table S9. Confidence metrics for the top-ranked predictions. Metrics include Confidence, pTM, ipTM, and iLDDT scores reported for AF3, Chai-1, and Boltz-2 across protein-aptamer complexes. Confidence, pTM, and ipTM values were taken directly from model outputs, and iLDDT scores were computed using OpenStructure on a 0–1 scale. Complex names in red indicate cases absent from the Boltz-2 training set.

Complex	Model	Confidence	pTM	ipTM	iLDDT
7lri	AF3	0.95	0.94	0.94	0.84
	Boltz-2	0.94	0.96	0.96	0.84
	Chai-1	0.91	0.95	0.90	0.78
7szu	AF3	0.81	0.83	0.81	0.91
	Boltz-2	0.96	0.93	0.92	0.93
	Chai-1	0.86	0.90	0.85	0.87
7v5n	AF3	0.89	0.88	0.88	0.75
	Boltz-2	0.97	0.98	0.98	0.91
	Chai-1	0.79	0.85	0.77	0.64
7zko	AF3	0.81	0.83	0.72	0.55
	Boltz-2	0.85	0.96	0.92	0.56
	Chai-1	0.87	0.90	0.86	0.45
7zqs	AF3	0.87	0.81	0.78	0.47
	Boltz-2	0.79	0.81	0.78	0.38
	Chai-1	0.71	0.79	0.69	0.31
8d29	AF3	0.86	0.85	0.84	0.89
	Boltz-2	0.97	0.96	0.96	0.92
	Chai-1	0.85	0.90	0.84	0.83
8bw5	AF3	0.67	0.76	0.59	0.49
	Boltz-2	0.85	0.94	0.89	0.67
	Chai-1	0.85	0.89	0.84	0.48
8tfd	AF3	0.72	0.86	0.69	0.13
	Boltz-2	0.86	0.95	0.86	0.23
	Chai-1	0.71	0.92	0.65	0.15
8tqs	AF3	0.94	0.91	0.89	0.88
	Boltz-2	0.93	0.97	0.96	0.87
	Chai-1	0.89	0.93	0.88	0.72
8zbf	AF3	0.41	0.52	0.35	0.38
	Boltz-2	0.79	0.93	0.93	0.27
	Chai-1	0.44	0.59	0.40	0.09
9gxh	AF3	0.58	0.82	0.52	0.16
	Boltz-2	0.91	0.97	0.96	0.26
	Chai-1	0.69	0.93	0.63	0.10

Table S10. ΔG_{bind} (kcal mol^{-1}) calculated over 20–100 ns MD time windows for the top-ranked AF3 predictions. The reported protein-aptamer complexes are 8BW5, 9GXH, and 8TFD, with aptamer structures corresponding to duplex/G-quadruplex, G-quadruplex, and stem-loop folds, respectively.

Complex	Window (ns)	AF3
8bw5	20–40	-20.60
	40–60	-40.26
	60–80	-34.46
	80–100	-32.59
8tfd	20–40	-49.88
	40–60	-45.61
	60–80	-91.00
	80–100	-86.48
9gxh	20–40	-49.48
	40–60	-74.29
	60–80	-85.83
	80–100	-62.29

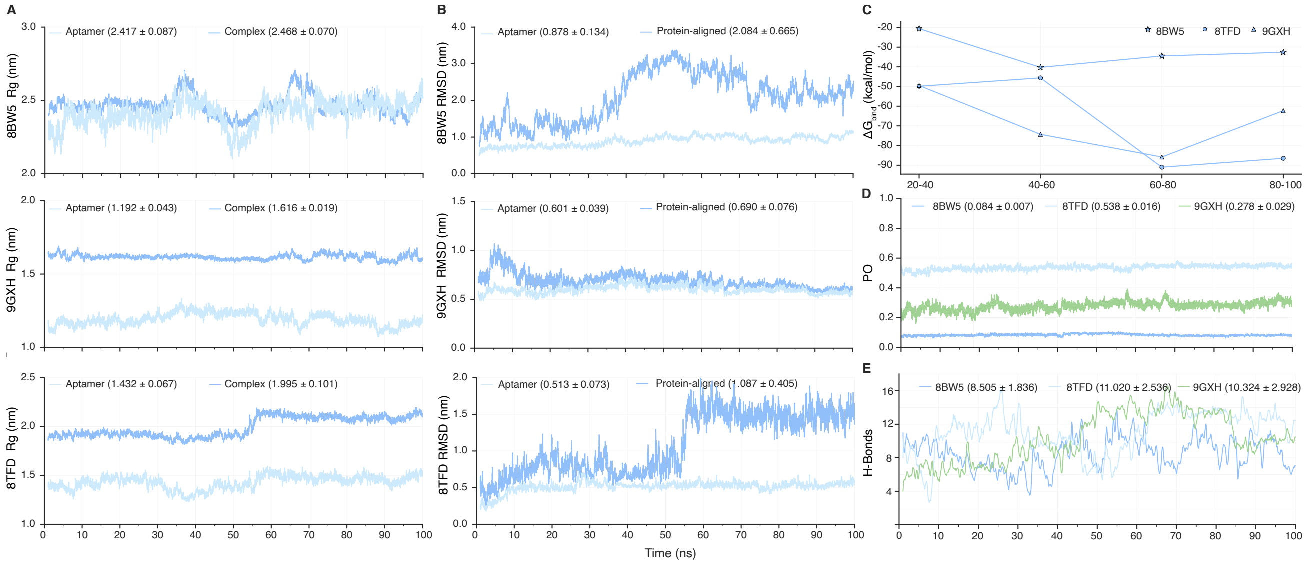

Fig. S15. Results from 100 ns MD simulations of the top-ranked AF3 predictions. The reported protein-aptamer complexes are 8BW5, 9GXH, and 8TFD, with aptamer structures of duplex/G-quadruplex, G-quadruplex, and stem-loop folds, respectively. (a) Rg profiles for the combined protein-aptamer complex (Complex) and the aptamer component within the complex (Aptamer). (b) RMSD profiles of the aptamer within the complex (Aptamer) and after protein-backbone alignment (Protein-aligned). (c) ΔG_{bind} over four consecutive 20 ns sub-windows within the 20–100 ns interval. (d) Pocket occupancy. (e) Number of hydrogen bonds. Values in parentheses indicate mean ± standard deviation over the analyzed trajectory.

Table S11. Aptamer structural classes and experimentally resolved ions in the benchmark complexes. "Included in ion-inclusion analysis" indicates whether the complex was included in the ion-inclusion prediction analysis. Complex names in red indicate cases absent from the Boltz-2 training set.

PDB ID	Aptamer structural class	Resolved ion(s)	Included in ion-inclusion analysis
7lri	duplex DNA	Ca^{2+}	Yes
7szu	ssRNA	Mg^{2+}, Na^{+}	Yes
7v5n	ssDNA	None	No
7zko	G-quadruplex ssDNA	K^{+}, Na^{+}	Yes
7zqs	ssDNA	Mg^{2+}	Yes
8d29	tertiary-structured ssRNA	K^{+}, Na^{+}	Yes
8bw5	duplex/G-quadruplex DNA	Na^{+}	Yes
8tfd	stem-loop ssDNA	None	No
8tqs	ssDNA	Mg^{2+}, Na^{+}	Yes
8zbf	ssDNA	Na^{+}	Yes
9gxh	G-quadruplex DNA	K^{+}	Yes
